# A Systematic Review and Meta-Analysis of Diagnostic and Prognostic Serum Biomarkers of Colorectal Cancer

**DOI:** 10.1371/journal.pone.0103910

**Published:** 2014-08-08

**Authors:** Zhongyu Liu, Yingchong Zhang, Yulong Niu, Ke Li, Xin Liu, Huijuan Chen, Chunfang Gao

**Affiliations:** 1 Anal-Colorectal Surgery Institute, Central Hospital of PLA, Luoyang, Henan, China; 2 Chengdu Municipal Centers for Disease Control & Prevention, Chengdu, China; 3 College of Life Science, Sichuan University, Chengdu, China; Centro di Riferimento Oncologico, IRCCS National Cancer Institute, Italy

## Abstract

**Background:**

Our systematic review summarizes the evidence concerning the accuracy of serum diagnostic and prognostic tests for colorectal cancer (CRC).

**Methods:**

The databases MEDLINE and EMBASE were searched iteratively to identify the relevant literature for serum markers of CRC published from 1950 to August 2012. The articles that provided adequate information to meet the requirements of the meta-analysis of diagnostic and prognostic markers were included. A 2-by-2 table of each diagnostic marker and its hazard ratio (HR) and the confidence interval (CI) of each prognostic marker was directly or indirectly extracted from the included papers, and the pooled sensitivity and specificity of the diagnostic marker and the pooled HR and the CI of the prognostic marker were subsequently calculated using the extracted data.

**Results:**

In total, 104 papers related to the diagnostic markers and 49 papers related to the prognostic serum markers of CRC were collected, and only 19 of 92 diagnostic markers were investigated in more than two studies, whereas 21 out of 44 prognostic markers were included in two or more studies. All of the pooled sensitivities of the diagnostic markers with > = 3 repetitions were less than 50%, and the meta-analyses of the prognostic markers with more than 3 studies were performed, VEGF with highest (2.245, CI: 1.347–3.744) and MMP-7 with lowest (1.099, CI: 1.018–1.187)) pooled HRs are presented.

**Conclusions:**

The quality of studies addressing the diagnostic and prognostic accuracy of the tests was poor, and the results were highly heterogeneous. The poor characteristics indicate that these tests are of little value for clinical practice.

## Introduction

Colorectal cancer (CRC) is one of the most common malignancies in developed countries [1]. The incidence of CRC in China was lower than that in the West but has increased in recent years [2] and has become a substantial cancer burden in China. The CRC mortality rate in China is 7.35/100,000 people, according to a retrospective survey on deaths caused by malignant tumors in China from 2004 to 2005[3]. Each year in the United Kingdom and the United States, there are approximately 32,000 and 160,000 new cases diagnosed, respectively, and approximately 500,000 new cases diagnosed worldwide [4]. Despite advances in dosing and scheduling of chemotherapy in both adjuvant and advanced settings, early detection of CRC is always over-emphasized [5].

The FOBT (fecal occult blood test) and colonoscopy are the traditional methods for CRC screening. Although the FOBT is non-invasive and cheap, the lower sensitivity of the results makes it unacceptable for promotion and popularization [6]. Although colonoscopy plus biopsy is the gold standard of colorectal cancer screening and diagnosis because of the invasive nature and intestinal discomfort of colonoscopy, more than half of patients do not want it [7]. Compared with these screening methods, tests of serum biomarkers are more convenient and less invasive and can be more acceptable as part of a routine physical examination [8], but most serum CRC markers still remain poor for most patients [9]. Although a number of serum markers of outcome in CRC have been reported [10], there has been no clear consensus as to their role, with many studies reporting conflicting results [11]–[13].

An important consideration is that a systematic review can highlight the underlying problems across individual studies and help identify the need for future research [14]. In the current paper, both of these aspects are addressed, and we hope that our findings will improve studies on CRC markers in the future.

## Materials and Methods

### Search strategy

The systematic search addressed articles with information on markers in serum to include or exclude the presence of CRC published from January 1950 to August 2012. To fulfill our selection criteria, the studies had to have been published as a full paper in English. Articles were identified by an electronic Medline and PUBMED search using the following keywords: ‘Colorectal’, ‘Colon’, ‘rectal’, ‘cancer’, ‘serum’ and ‘marker’ (See Appendix 1 in Materials S1 for the key words and corresponding “associated words”; see Appendix 2 for the details of search strategy). In the current study, duplicates from Medline and EMBASE were deleted automatically and manually with Reference Manager Version 11 (Thomson Reuters, New York, NY, USA).

### Inclusion and exclusion criteria

For diagnostic marker(s), the meta-analysis focuses on the sensitivity and specificity of a marker, and the most basic requirement is a 2×2 table of outcome by marker index test to calculate the two values. A brief overview of the criteria for a diagnostic marker is the following:

1. The **original** article is **in English** and about **diagnostic serum marker(s)** of only **primary colorectal cancer** (**CRC, colon or rectal cancer**).
2. There is enough information to **directly or indirectly** construct **a 2×2 table(s)** of outcome by the marker(s) index test.
3. The **gold standard** (reference standard) for the diagnosis of **CRC, colon or rectal cancer** is based on **clinical histopathology**.
4. **Only patients (CRC, colon or rectal cancer) versus control (healthy population) are examined.**

Auxiliary information such as study design and cut-off values (see Table S3 of our manuscript) is not very important for quantitative synthesis of effect sizes of a diagnostic marker. We summarized study designs for studies with the following designs: case-control, retrospective case-control, prospective cohort, nested case-control, prospective nested case-control, cohort, prospective cohort and cohort of consecutive patients (see Table S3 for details).

For prognostic marker(s), the study must provide time-to-event data, and the meta-analysis focuses on hazard ratio (HR) and its confidence interval (CI)

1. An **original** paper based on a **primary CRC, colon or rectal cancer** in **English** had to provide a quantitative result or give tabulated individual patient data **(IPD)** [15] to assess the ability of one or more **prognostic serum markers.**
2. The study should provide sufficient data to (re)construct a 2×2 table to estimate the marker's prognostic accuracy or the log of the **hazard ratio (HR)** and its precision (the v**ariance or standard error (SE)**) or the HR and its **confidence interval (CI)**.

In addition to the above 2 items, the rest of the items are the same as items 3 and 4 for diagnostic markers.

From papers classified as ‘relevant,’ information was extracted on the tumor marker used, the clinical area of application, the age range of patients, stage of disease, whether the outcome was overall survival (OS) or disease-free survival (DFS), and the cut-off level of the marker (See Table S5 of our manuscript for the details).

Two stages were needed to include or exclude the candidate articles. The first batch of reviewers, who were trained in advance, assessed the titles and abstracts, and then, the second independent batch of reviewers, who were trained in advance, assessed the full articles to assure that no relevant articles were excluded. Inclusion or exclusion, as well as data extraction for any paper, was implemented by at least two independent reviewers, and if the extracted data were not the same, conflicts were resolved by reaching a consensus. 1) If more than one marker was used in a given study, the relevant data for each eligible marker was individually extracted. 2) If one marker had multiple functions (i.e., one marker for one disease is used for screening, diagnosis, prognosis and/or monitoring), the datasets corresponding to the multiple functions were extracted separately. 3) If there were multiple markers and diseases addressed in one study, only the relevant data from the marker(s) corresponding to each disease of interest to the author(s) was extracted.

### Data extraction

From papers classified as “relevant,” information was extracted on the study characteristics, the participant characteristics, the type of reference test used to confirm the presence or absence of colorectal cancer, the tumor marker used, the clinical area of application, the age range of patients, the stage of disease, whether the outcome was overall survival (OS) or disease-free survival (DFS), and the cut-off levels as well as how these levels were determined. Some of the studies had several different cut-off levels, and we only took the one closest to the cut-off corresponding with 95% specificity (avoiding false positives as much as possible) [16] 1) For diagnosis-related papers, the data extraction and methodological quality assessment of each included study were generally performed simultaneously. Whiting et al. (2003) proposed a set of criteria for the Quality Assessment of Diagnostic Accuracy Studies (QUADAS) that applies well to diagnostic marker studies [17]. Additional information to be extracted included the number of patients and controls and the numbers of true positives (TP)/false positives (FP)/true negatives (TN)/false negatives (FN), which are mandatory. In addition, the sensitivity and specificity, the 95% confidence intervals (CIs), the overall accuracy, the positive predictive value (PPV = TP/(TP+FP)), the negative predictive value (NPV = TN/(TN+FN)), the positive likelihood ratio (LR+), the negative likelihood ratio (LR−), and the diagnostic odds ratio (DOR) of the tumor markers were optional extracted information. If a study lacked the mandatory information, we calculated the TP/FP/TN/FN and filled in the blanks in the table. 2) For prognosis-related papers, Altman et al. (2012) proposed reporting recommendations for tumor marker prognostic studies (REMARK) [18] that apply well to prognostic marker studies. The data extraction and conversions for prognostic markers were much more complex than for diagnostic markers because prognostic markers provide time-to-event data. Meta-analyses of this type of marker often require one of two types of data, i.e., the log of the hazard ratio (HR) and its precision (the variance or standard error (SE)) or the HR and its confidence interval (CI). For major prognostic marker studies, the two types of data cannot be extracted directly. Paramar and colleagues [19] presented a series of simple methods to extract the relevant data from publications with the aim of performing a meta-analysis of survival-type data. The methods focus on approaches for extracting these data from publications and are illustrated throughout this publication with real examples. Riley and co-workers (2003) [20] summarized 11 methods (Appendix 3) that are available for directly or indirectly estimating these data and the approximate normal loge (HR) distribution for large samples. In addition, Tierney et al. [21] provided step-by-step guidance for how to calculate an HR and the associated statistics for individual trials, according to the information presented in the trial report. In our study, an R package was developed based on the methods of Paramar and colleagues [19] and was applied to indirectly or directly calculate the HR and its CI.

### Statistical analysis and data synthesis

The systematic review process followed the guidelines published by the NHS Centre for Reviews and Dissemination and had an overall objective of maintaining breadth, synthesizing the evidence qualitatively and then, only where appropriate, using quantitative methods [22], [23].

### Diagnostic serum markers

Meta-analysis of diagnostic test accuracy presents many challenges. Even in the simplest case, when the data are summarized by a 2×2 table from each study, a statistically rigorous analysis requires hierarchical (multilevel) models that respect the binomial data structure. In the current study, the forest plots of sensitivity and specificity estimates and their 95% CIs were constructed from every study using MetaDiSc software (version 1.4) [24], with the heterogeneity of the accuracy estimates assessed with the I^2^ statistic [25]. The summary estimates of sensitivity and specificity were calculated using the package Metandi for STATA 11 statistical software (STATA Corp, College Station, TX) [26] (Metandi requires either Stata 10 or above). We also adopted a command, metandiplot, to simplify the plotting of graphical summaries of the fitted model, namely, the summary receiver operating characteristic (SROC) curve and the prediction region and also to plot the summary point and its confidence region.

It has been argued that diagnostic accuracy test may be particularly susceptible to publication bias [27]. Simulation studies have, however, indicated that the effect of publication bias on meta-analytic estimates of the Diagnostic Odds Ratio (DOR) is not likely to be large, and its assessment in reviews of test accuracy is complex [28]. An alternative approach uses funnel plots of (natural logarithm (ln) DOR) vs (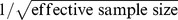) and tests for asymmetry using related regression or rank correlation tests [28]. It should be noted that the power of all statistical tests for funnel plot asymmetry decreases with increasing heterogeneity of DOR.

### Prognostic serum markers

The hazard ratio (HR) was used to measure the impact of the expression of individual biomarkers on prognosis. From papers classified as ‘relevant’, information was extracted on the tumor marker used, the clinical area of application, the age range of the patients, the stage of disease, whether the outcome was overall survival (OS) or disease-free survival (DFS), and the cut-off level of the marker. OS, DFS, or unclear were recorded to classify the outcome of a marker, where available, and separated according to whether they had been analyzed by univariate or multivariate analysis. Disease-specific survival (DSS) was included under OS, and distant disease-free survival (DDFS) and metastasis-free survival (MFS) were included under DFS. For both OS and DFS, the following were recorded (where available): whether the marker for analysis had a significant association with survival, the hazard ratio (HR), the 95% confidence intervals (CI), the p value for the factor, whether the p value was exact, and whether the survival had been analyzed by univariate and/or multivariate analysis. If multivariate analysis had been performed, other factors included in the model were also recorded. Because the estimate measure of HR varied, we converted the different statistics into the HR, 95% CI, and its variance, which were more accurate and united. After obtaining the basic statistics, a sequential process based on the appropriate command in STATA version 10 (Stata Corporation, College Station, TX, USA) was implemented to count the pooled HR value. The process followed the research of RD Riley [20].

Pooled estimates of the HRs were obtained using both fixed-effect and random-effect meta-analyses using the inverse-variance weighting method. Statistical heterogeneity between studies was assessed using the among-study variance (s2) and the statistic I^2^ [25]. We conducted heterogeneity χ2-tests, and if the assumption of homogeneity of individual HRs had to be rejected, we used a random-effect model in place of a fixed-effect model. By convention, an observed HR>1 implied a worse prognosis for the group with positive marker expression. We performed a meta-analysis of prognostic test accuracy using the metan command in STATA. Publication bias refers to the phenomenon of studies with uninteresting or unfavorable results being less likely to be published than those with more favorable results [29]. If a publication bias exists, then the published literature is a biased sample of all studies on a topic, and any meta-analysis based on it will be similarly biased. Funnel plots are commonly used to investigate publication and related biases in meta-analyses [30]. The metabias function in STATA performs the Begg and Mazumdar [31] adjusted rank correlation test for publication bias as well as the Egger et al. [32] regression asymmetry test for publication bias. As options, it provides a funnel graph of the data or the regression asymmetry plot. The Begg adjusted rank correlation test is more popular in common applications for publication bias analysis, and it is used to estimate the publication bias in our study. The “trim and fill” method [33] was implemented to explore the possible nature of studies “missed” in the review and to attempt to estimate the “true” relative risk estimate accounting for publication bias. The command metatrim in STATA is used to implement the Duval and Tweedie nonparametric “trim and fill” method.

## Results

### Searching results

**Figure 1:**
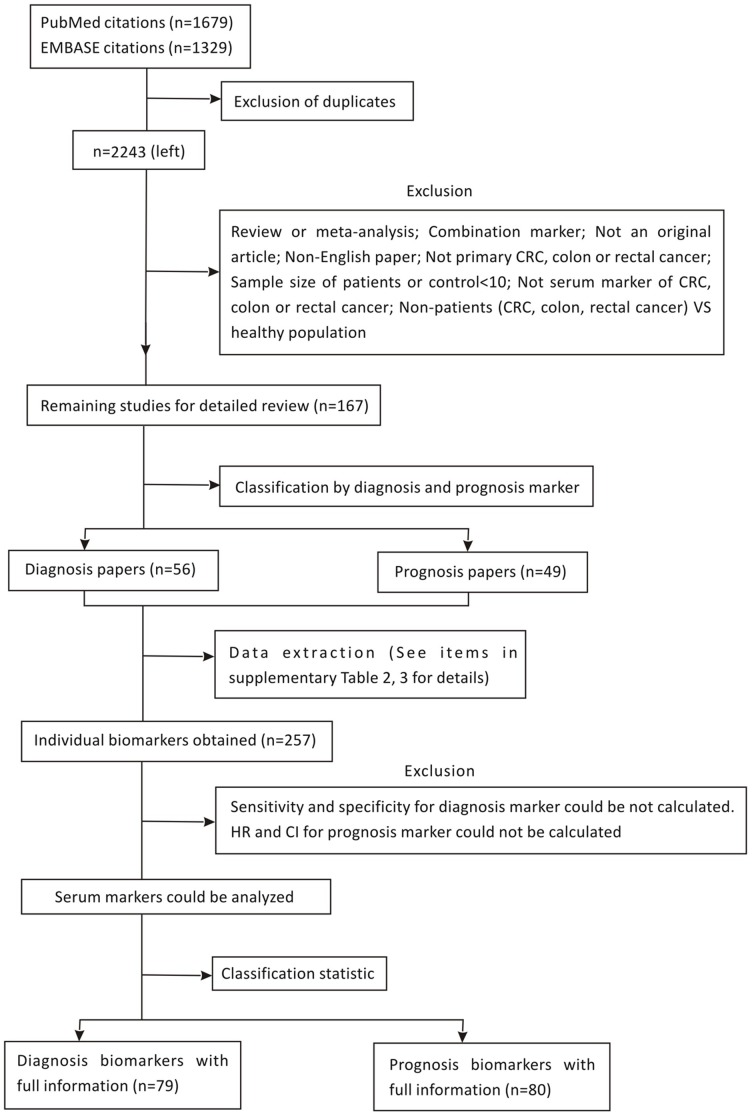
The flowchart of the selection of the relevant articles.

In total, 2243 articles were obtained from the two databases, of which 153 articles reporting on 114 CRC serum diagnostic and/or prognostic markers (Table S1) were considered as relevant according to the first two reviewers. A total of 105 papers (Appendix 4) were related to diagnosis, whereas 49 (Appendix 5) were prognosis papers. Furthermore, 23 of the relevant papers include both diagnosis and prognosis. In these studies, a total of 257 individual tumor markers were obtained. Papers indicating related studies in the specific area were studied further to seek more relevant results. The process of retrieving and reserving papers and the results are shown in Figure 1.

### Tumor Markers Identified Overall and Within Each Clinical Area

#### Assessment of study quality and Investigated diagnostic serum markers

**Figure 2:**
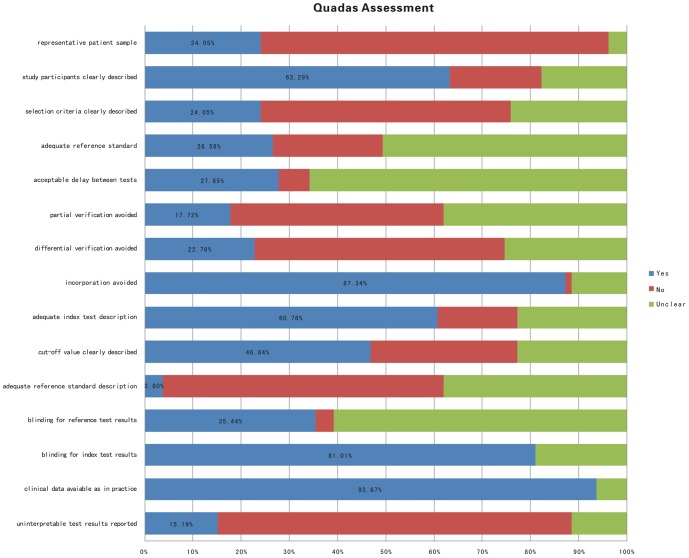
Summary of quality of the included studies, according to the QUADAS criteria (see Table S2 for details).

The quality of diagnosis papers was assessed by using the QUADAS system [11]. The methodological quality of the studies with a focus on the objective of this review was generally poor and are shown in Figure 2, with specific details in Table S2 (references to these studies are prefaced by a ‘D’ and are listed in Appendix 4 in Materials S1). Of the studies, 12 papers were designed using a prospective cohort study. The rest of studies used case-control methods. Therefore, verification bias inevitably appeared in those studies. Verification bias is the result of identifying experimental groups by the gold standard reference test of a disease or condition, such as cancer, whereas the control group is presumed to be free of this condition, but this is not verified by the gold standard reference test, which inflates sensitivity and decreases specificity [34]–[36]. Moreover, most studies did not have an adequate description of the patient-selection procedure, the characteristics of the study participants, the reference standard, and the used cut-off value of the marker. The time between the index test (marker) and the reference test as well as the availability of other clinical data (as is commonly encountered in practice) were also poorly reported.

Table S3 provides a complete summary of the performance of all markers across the included studies. In total, 92 serum markers were identified, and only a few markers are frequently reported. Of those markers, 73 markers are only reported one time. The most frequently evaluated serum marker was CEA (42 repetitions) followed by CA19-9 (24), CRP (9), CA-50 (7), CA72-4 (7), and VEGF (7) (Table 1). Some reviews may not result in useful summary estimates of sensitivity and specificity, for example, because of substantial variability in the individual study estimates or because the number of the relevant studies corresponding to a marker is less than three. Several methods of meta-analyzing diagnostic accuracy data have been proposed, of which, two are statistically rigorous: the hierarchical summary receiver operating characteristic (HSROC) model [37] and the bivariate model [38]. In current systematic review, the summaries of the diagnostic accuracy of those markers, respectively assessed by the hierarchical summary receiver operating characteristic (HSROC) curve [39] (study number>three) and the forest plot of meta-analysis (study number >2), are shown in Table 1. CEA is the most frequently studied biomarker based on the extracted biomarker information. In total, there are 42 papers presenting the diagnostic results for CEA. The CEA studies included 8861 individuals, of which 5361 were patients, and the remaining 3500 individuals were controls. The cut-off value ranged from 2.40 ng/ml to 10.0 ng/ml. The sensitivity and specificity ranged widely from 25.55% to 97.22% and 54.40% to 100.00%, respectively.

**Table 1:** Results of the meta-analysis of the diagnostic markers for colorectal cancer.

											Results adjusted for publication bias by the “trim and fill” method			
No	Marker Name	Full Name	No of studies	Reference ID	Pooled Sensitivity(95% CI)	Pooled Specificity(95% CI)	Heterogeneity(sen/spe p values)	I-square(sen/spe)	HSROC Plot	Forest plot of Sensitivity and Specificity	Publication bias (p Value)	Model	Studied added	Filled and pooled OR(95%CI)
1	CEA	carcinoembryonic antigen	42	d1–d26,d57–d72	0.461 (0.448–0.474)	0.892 (0.882–0.902)	0.000/0.000	83.1%/81%	Y	Y	0.019	random	8	1.354(0.871–1.838)
2	CA19-9	carbohydrate antigen 19-9	24	d1,d3,d7,d8,d9,d10,d13,d16,d18,d20,d25,d27,d57–d60,d62,d63,d65,d66,d69,d70,d71,d73	0.300 (0.283–0.318)	0.928 (0.915–0.940)	0.000/0.000	79.3%/60.1%	Y	Y	0.301	random	3	1.351(0.538–2.165)
3	CA242	cancer antigen 242	10	d16,d24,d59,d61,d63,d67,d69,d70,d71,d74	0.391 (0.368–0.413)	0.884 (0.864–0.901)	0.000/0.000	83.8%/91.3%	Y	Y	0.904	random	1	1.378(0.289–2.486)
4	CRP	C Reactive of protein	9	d28,d29,d30	0.326 (0.302–0.350)	0.738 (0.722–0.754)	0.312/0.008	14.7%/61.5%	Y	Y	0.491	fixed	1	−1.302(−1.433–−1.172)
5	VEGF	vascular endothelial growth factor	7	d22,d26,d31,d32,d75,d76,d77	0.562 (0.525–0.599)	0.806 (0.760–0.847)	0.000/0.000	95.3%/91.2%	Y	Y	0.026	random	1	1.269(−0.513–3.051)
6	CA-50	cancer antigen 50	7	d16,d17,d24,d59,d67,d78,d79	0.387 (0.343–0.431)	0.777 (0.743–0.809)	0.000/0.000	83.1%/97.2%	Y	Y	0.066	random	2	−0.064(−1.032–0.904)
7	CA72-4	cancer antigen 72-4	7	d1,d9,d10,d18,d62,d63,d71	0.299 (0.270–0.330)	0.957 (0.941–0.970)	0.000/0.902	85.6%/0	Y	Y	0.754	random	0	1.330(−0.170–2.831)
8	IGFBP-3	insulin-like growth facter binding protein 3	5	d33,d34,d35,d36	0.202 (0.187–0.217)	0.795 (0.780–0.809)	0.000/0.004	82.3%/72.6%	Y	Y	0.007	random	1	−1.151(−2.158–−0.144)
9	TAG-72	Tumor-associated glycoprotein-72	5	d9,d11,d12,d37	0.427 (0.387–0.468)	0.961 (0.942–0.976)	0.280/0.000	21.1%/81.4%	Y	Y	0.406	random	0	1.609(−1.547–4.765)
10	IGF-1	insulin-like growth facter 1	4	d33,d34,d35,d36	0.220 (0.200–0.242)	0.780 (0.760–0.798)	0.002/0.001	80.2%/81.8%	Y	Y	0.039	random	0	−0.927(−1.780–−0.075)
11	P53	P53	3	d81,d82,d83	0.231 (0.188–0.278)	1.000 (0.966–1.000)	0.026/1.000	72.6%/0	N	Y	N/A	N/A	N/A	N/A
12	CA125	cancer antigen 125	2	d1,d20	0.180 (0.142–0.224)	0.950 (0.919–0.972)	0.663/0.777	0/0	N	Y	N/A	random	0	1.385(0.837–1.934)
13	c-erbB-2	c-erbB-2 protein	2	d2,d38	0.320 (0.241–0.409)	0.633 (0.558–0.704)	0.000/0.000	95.8%/93.6%	N	Y	N/A	fixed	0	−0.866(−7.235–5.503)
14	TIMP-1	tetramethylbenzidine	2	d1,d80	0.454 (0.421–0.488)	0.952 (0.925–0.971)	0.000/0.916	98.5%/0	N	Y	N/A	random	1	1.690(−1.725–5.104)
15	M2-PK	M2-PK	2	d84,d85	0.518 (0.460–0.576)	0.932 (0.847–0.977)	0.006/0.527	86.7%/0	N	Y	N/A	fixed	0	3.977(3.028–4.926)
16	TPA-M	tissue polypeptide antigen	2	d5,d19	0.701 (0.647–0.752)	0.882 (0.810–0.934)	0.897/0.000	0/96.2%	N	Y	N/A	fixed	0	3.317(2.603–4.031)

Notes: If the number of is more than three, the HSROC Plot and forest plot can be drawn, if the number of studies is more than two, only the forest plot can be drawn. Reference IDs to these studies are prefaced by a ‘D’ and listed in Appendix 4 in Materials S1. Y denotes Yes; N denotes No; N/A denotes not applicable, which means the value is not available. If the number of the studies is less than three, the p value of publication bias cannot be calculated. In addition, the false positive rate of marker P53 is zero, and then the odd ratio (OD) cannot be calculated, so all values are not applicable.

**Figure 3:**
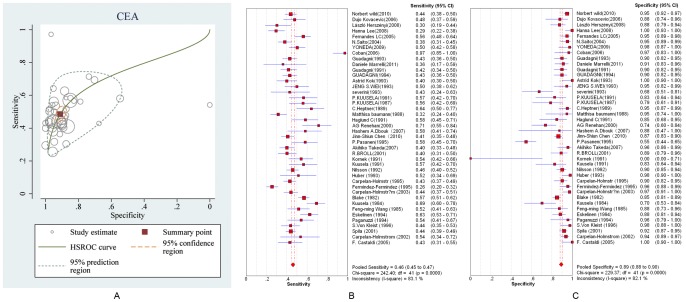
The ROC and forest plots of summary estimates of sensitivity and specificity of diagnostic marker CEA. A is the ROC plot of the hierarchical summary estimates of sensitivity and specificity for CEA with 95% confidence and prediction ellipses. B and C are forest plots of sensitivity and specificity of the diagnostic marker CEA for colorectal cancer plotted with a HSROC model. The size of the squares in B and C are proportional to the study size and weight for each study. The rhombus represents the pooled estimates, which are 0.461 (CI: 0.448–0.474) and 0.892 (CI: 0.882–0.902) for specificity and sensitivity, respectively.

Figure 3 A presents hierarchical summary estimates of sensitivity and specificity for CEA after back-transformation to ROC axes. Furthermore, it shows the 95% confidence ellipse around the mean values of sensitivity and specificity for CEA and a 95% prediction ellipse for the individual values of sensitivity and specificity. The ellipse around the summary or mean estimate of sensitivity and specificity marks the region containing likely combinations for which the mean value of sensitivity and specificity is small. The 95% prediction ellipse is wider and indicates more uncertainty as to where the likely values of sensitivity and specificity might occur for individual studies. Figures 3 B and C separately present the forest plots of the specificity and sensitivity of the diagnostic marker CEA for colorectal cancer with individual study estimates of the sensitivities and specificities and the 95% CIs as a random-effects model. The simple summary estimates of the sensitivity and specificity of CEA for colorectal cancer were 46.1% (95% CI: 44.8–47.4%) and 89.2% (95% CI: 88.2–90.2%), respectively. The HSROC model produced the same summary estimates of sensitivity and specificity with almost exactly equal CIs (48.5% (95% CI: 44.8–52.3–46.7%) and 91.1% (95% CI: 88–93.0%), respectively) that take into account the heterogeneity beyond chance between studies (random-effects model). For the remaining serum markers for CRC, the pooled sensitivities and specificities with their CIs are, respectively, listed in the 6^th^ and 7^th^ columns in Table 1, but the HSROC plots and forest plots are presented in Appendix 6 in Materials S1 because of article length limits. Publication bias analyses were implemented for the prognostic markers with more than three repetitions in studies. The results are shown in the 12th–15th column in Table 1, and the characteristics of those makers are listed in Table S3. The corresponding forest plots and funnel plots are shown Appendix 7 in Materials S1. The results indicate that the publication bias exist for almost all diagnostic markers.

#### Assessment of study quality and Investigated prognostic serum markers

The scores of all prognostic studies by REMARK [18] are shown in Table S4. The scores of these studies ranged between 16 and 19. Table S5 provides a complete summary of the performance of all prognostic markers for CRC, across the included studies. In total, 41 serum prognostic markers were identified, and only a few markers were frequently reported. Of those markers, 22 markers were only reported one time, 13 markers were reported twice, and only 10 markers were reported more than three times. The most frequently evaluated serum prognostic marker was CEA (34 repetitions) followed by CA19-9 (10), VEGF (9), MASP-2 (6), CRP (5), TIMP-1 (4), YKL-40 (3), MMP-7 (3), PAI-1 (3), and suPAR (3). The prognostic markers with more than three repetitions were chosen for the meta-analysis and publication bias analysis using STATA (10 version) software, and the summaries are given for each marker in Table 2.

**Table 2:** The results of meta-analysis of prognostic makers for colorectal cancer.

						Meta-analysis				Results adjusted for publication bias by the “trim and fill” method			
No	Maker name	Full name	No.of studies	Reference IDs	Outcome	Pooled HR(95%CI)	Heterogeneity(p Value)	I-square	Model	Publication bias (p Value)	Model	Studied added	Filled and pooled HR(95%CI)
1	CEA	carcino-embryonic antigen	12	P2 P3 P5 P9 P10 P11 P12 P13 P16 P20 P23 P26	DFS	1.624(1.290–2.043)	0.000	0.842	random	0.000	random	3	1.346 (1.083–1.671)
			14	P1 P2 P3 P7 P9 P13 P14 P15 P16 P17 P21 P22 P23 P25	OS	1.453(1.267–1.666)	0.000	0.853		0.000	random	7	1.166(1.018–1.336)
			8	P4 P6 P8 P18 P19 P24 P27 P28	unclear	2.208(1.479–3.297)	0.000	0.913		0.000	random	1	2.073(1.410–3.047)
			34		overall	1.513(1.391–1.645)	0.000	0.89		N/A	N/A	N/A	N/A
2	CA19-9	carbohydrate antigen 19-9	5	P5 P11 P12 P20 P26	DFS	**1.572(0.808–3.059)**	0.077	0.526	random	0.077	fixed	0	1.711(1.135–2.579)
			4	P7 P15 P20 P30	OS	1**.782(0.851–3.729)**	0.000	0.853		0.000	random	0	**1.782(0.851–3.729)**
			1	P29	unclear	1.750(1.288–2.377)	N/A	0		N/A	N/A	N/A	N/A
			10		overall	1.745(1.200–2.538)	0.001	0.695		N/A	N/A	N/A	N/A
3	VEGF	vascular endothelial growth factor	6	P10 P17 P18 P31 P32 P33	OS	2.597(1.404–4.802)	0.000	0.875	random	0.000		2	1.106(1.053–1.162)
			3	P24 P33 P34	unclear	**1.521(0.462–5.004)**	0.029	0.717		0.029		0	**1.521(0.462–5.004)**
			9		overall	2.245(1.347–3.744)	0.000	0.838		N/A	N/A	N/A	N/A
4	MASP-2	mannan-binding lectin-associated serine protease-2	3	P35 P36	DFS	1.451(1.264–1.666)	0.881	0	fixed	0.881	fixed	2	1.400(1.176–1.666)
			3	P35 P36	OS	1.489(1.195–1.856)	0.773	0		0.773	fixed	2	1.400(1.244–1.575)
			6		overall	1.462(1.300–1.643)	0.977	0		N/A	N/A	N/A	N/A
5	CRP	C-reactive protein	3	P32 P37 P39	OS	**2.498(0.977–6.387)**	0.000	0.896	random	0.000	random	2	**1.130(0.532–2.400)**
			2	P19 P38	unclear	1.897(1.570–2.293)	0.971	0		0.971	fixed	0	1.897(1.570–2.293)
			5		overall	1.977(1.282–3.050)	0.000	0.907		N/A	N/A	N/A	N/A

Note: The reference IDs for these studies are prefaced by a ‘P’ and listed in Appendix 5 in Materials S1. Bold texts in the boxes indicate that the pooled HRs are not significant (because the 95% confidence interval for the HRs overlap 1); OS: overall survival; DFS: disease-free survival; HR: hazard ratio; CI: confidence interval; random: random-effect model; fixed: fixed-effect model; N/A: not applicable.

**Figure 4:**
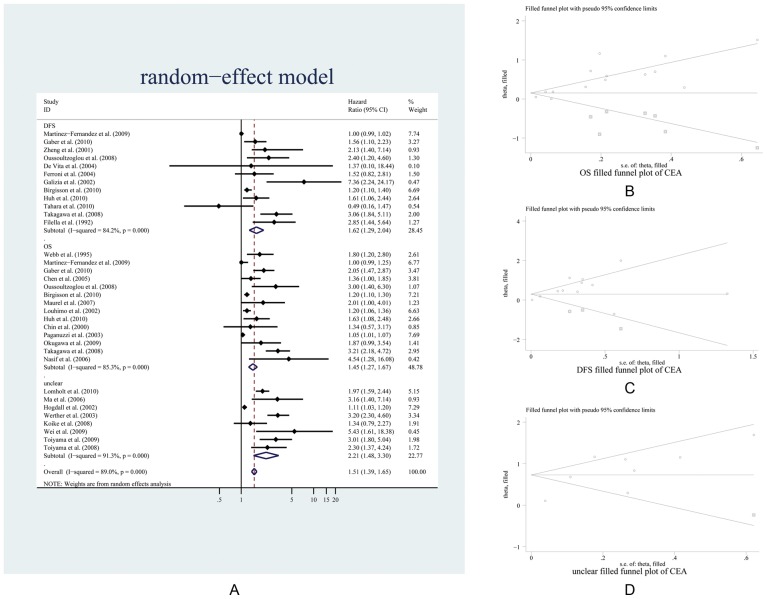
Meta-analysis plots of the progression-free and overall survival hazard ratios in individual trials. A is the forest plot and B, C, and D are the “filled” funnel plots of OS, DFS, and the unclear group, respectively. The meta-analysis displayed a significant effect in favor of a high volume. The pooled and filled results are presented in Table 2.

The most frequently reported prognostic marker for CRC is CEA. The CEA studies included 5792 patients, of which 3856 patients had positive results for the CEA marker, whereas 1936 patients were negative. The cut-off values ranged from 2.7 ng/ml to 10.0 ng/ml. The median patient age across all trials was between 47.74 and 73 years, with an age range of 31—90 years. All patients had histologically or cytologically confirmed CRC, colon or rectal cancer, as the primary diagnosis. There are 28 articles related to CEA and the prognosis outcome of the patients, of which 6 articles studied both the overall survival (OS) and disease-free survival (DFS). There are 9 articles that do not state whether they studied the OS or DFS; we defined these as “unclear” (Table 2). A summary of the individual trials and overall pooled results from the primary analysis of the overall survival is shown in Figure 4. According to the outcomes (OS, DFS and unclear), the CEA was classified into three subgroups, and the three subgroup datasets were separately submitted to the meta-analysis and publication bias analysis. As a result, the pooled HRs with 95% CIs of OS, DFS, and unclear subgroups were 1.624 (1.290–2.043), 1.453 (1.267–1.666), and 2.208 (1.479–3.297), respectively, and the overall HR (CI) from the three combined subgroups was 1.513 (1.391–1.645) (Figure 4 A). After analysis of the publication bias by the “trim and fill” method, the OS, DFS, and unclear subgroups were added with three, seven, and one “missing” studies (Figure 4 B C and D and Table 2), respectively. The adjusted HRs with the 95% CIs for the three subgroups were 1.346 (1.083–1.671), 1.166 (1.018–1.336) and 2.073 (1.410–3.047), respectively. In contrast, all adjusted HRs were relatively smaller than the unadjusted HRs (Table 2, panel CEA). Likewise, the same methods of meta-analysis and publication bias analysis were implemented for the remaining prognostic markers with more than three repetitions in studies on CRC. The results are shown in Table 2, and the characteristics of those makers are listed Table S5. The corresponding Forest plots and funnel plots are shown Appendix 8 in Materials S1.

## Discussion

### Appraisal of the Systematic Review

In our study, we performed a systematic review and meta-analysis for all of the published CRC serum biomarkers. Through the investigation, we searched 114 serum biomarkers (for diagnosis 92, for prognosis 41), of which 20 biomarkers can both act as diagnosis and prognosis markers. Most of the markers have been published only once, and the most frequently reported top three markers for diagnosis are CEA (42 studies), CA19-9 (25 studies), and CA242 (10 studies), and for prognosis, they are CEA (34 studies), CA19-9 (10 studies), and VEGF (9 studies). For the diagnosis markers that were studied more than twice, we used the HSROC model and meta-analysis approach for the sensitivity and specificity correlation analysis. The results suggested that almost all of the pooled sensitivities of the diagnosis markers were less than 50% and followed by significant heterogeneity. Publication bias exists for major diagnostic serum CRC markers by an alternative approach using funnel plots of (natural logarithm (ln) DOR) vs (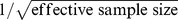) [28]. Likewise, meta-analyses and publication bias analysis were implemented for the prognostic markers with more than three repetitions in studies. The range of all of the pooled HRs is from 1 to 2, which indicates there will be no survival rate differences between the positive and negative patients. According to our analysis, we may explain why those reported diagnostic and prognostic markers of CRC are not suitable for clinical applications. Because most of the pooled sensitivities of the diagnosis markers were less than 50%, and the heterogeneity was significant, and the pooled HRs of the prognosis markers were greater than 1 and less than 2.

The ideal study sample for a test accuracy study is a consecutive or randomly selected series of patients in whom the target condition is suspected, or for screening studies, the target population. There are two basic types of test accuracy studies: cohort studies and case-control studies. Both diagnostic and prognostic studies included in the current systematic review predominantly belong to the case-control design type, which is liable to bias [40]. Diagnostic or prognostic tests perform differently in different populations [41], [42], It is important to clearly define the population of interest. In our systematic review, the study population is limited to primary CRC.

### Analysis of potential reasons for publication and heterogeneity observed

A potential source of bias (i.e., publication bias) is whether all relevant studies have been identified, and a small number of part-published studies may have been omitted. From table 1 and table 2, both diagnostic and prognostic studies have publication bias. In our search strategy, although we included as many key words and relevant works in our initial search strategy as possible, we acknowledge the possibility that this review was not exhaustive, reflecting publication and reporting bias. The reasons for “missing” papers may include the following: (1) the key words and relevant words related to CRC may not be fully comprehensive; (2) we did not search all of the literature databases (only EMBASE and PubMed were searched, but we believe these two databases include the majority of candidate papers); (3) we did not include non-English language papers because of the difficulties in translation, and this may have introduced bias if statistically or clinically significant studies were more likely to be (re)written for publication in an English language journal [43]; (4) a few articles were found in the two databases, but they could not be downloaded, in part because they were published too long ago or the journal that published those articles is too unpopular; (5) some papers did not provide a complete report of the data in the original article. Despite these concerns, the papers included in our study account for the vast majority of all papers relevant to CRC, and we believe that the final results are representative of the significance.

Another potential source of bias specific to this study is that of overlapping datasets. In our research, we minimized this bias by excluding such datasets, replacing these with only the most recent study.

Heterogeneity between studies may represent a further potential source of bias, but it is indispensable for any meta-analysis that potential sources of heterogeneity are examined, and variability beyond chance can be attributed to between-study differences in the selected cut-point for positivity, in patient selection (such as: severity of illness, age, gender and etc.) and clinical setting (such as dose, timing or duration of treatment), in the type of test used, in real variation in the treatment effect, in the type of reference standard, or any combination of these factors. In addition, heterogeneity in study results can also be caused by flaws in study design [44]. In reviews of studies on the prognostic accuracy of tests, heterogeneity may be influenced by duration of follow-up or the reliability of outcome measures [45]. To overcome the problem of heterogeneity, we provide some suggestions to improve study design standards and design large prospective studies to answer pre-specified questions of clinical interest. Weakness of reporting, analysis and presentation of results was frequently apparent throughout the evaluation of the selected papers. The presentation of survival analyses was particularly poor and the HR and its CI were often not reported directly. Accordingly, we can promote better reporting. We should conduct large prospective multi-center studies, and the multi-disciplinary teams can collaborate to seek consistency in cut-offs, adjustment factors, outcomes, analysis, measurement methods and other relevant variables.

### Interpretation of the diagnostic serum markers

For the diagnostic markers of CRC, various aspects, such as the diverse populations used (different age, origin, “normal,” or diseased controls), the diverse number of markers evaluated (single versus combined markers), and the use of different cut-off points for the same marker, result in an order of magnitude range of sensitivities and specificities reported for the various markers. Moreover, the majority of the markers (73/91, 80.2%) were evaluated in only one study (Table S3). Interpretation of many studies is further limited by the selection of cases and controls because only case-control studies may overestimate the sensitivity and specificity [46]–[48]. In case-control studies, the case group of patients may include an order of magnitude range with different pathological grades, ages, genders, regions and ethnicities. On the other hand, the controls had often not undergone colonoscopy. These control groups most likely included a substantial proportion of adenoma carriers because the prevalence of adenomas among older adults is estimated to be approximately 20% to 30% [49]–[51]. In CRC marker studies, the patient group should be compared with multiple control groups, such as other types of cancer and other intestinal diseases, advanced adenoma cases and a normal healthy population. Without these comparisons, the marker cannot be exactly correlated to CRC, and the specificity may be inaccurately estimated in such studies. In addition, the effect of the value of a new CRC serum marker is not reliable because of the lack of double-blind randomized clinical trials. Another concern refers to the comparability of results across studies given the potential differences in serum collection, processing, and storage methods, and uncertainties in the stability of several biomarkers. Information on these issues is very limited. All of the above-mentioned factors may cause variation in the results for markers of CRC, leading to imprecisely pooled results in the meta-analysis.

### Interpretation of the prognostic serum markers

Prognostic research has, to date, received much less attention than research into therapeutic or diagnostic areas, and an evidence-based approach to the design, conduct and reporting of primary studies of prognostic markers is needed [52]. Reviews have demonstrated that primary prognostic studies are often of poor quality [53]. Furthermore, synthesis of prognostic studies is a relatively new and evolving area in which the methods are less well developed than for reviews of therapeutic interventions or of diagnostic accuracy and available reviews have often been of poor quality [54]–[57]. For prognostic markers, apart from the duration of follow-up, the various aspects leading to heterogeneity observed are almost similar to those for diagnostic markers. Throughout the evaluation of the 49 selected papers, weaknesses in the analysis, reporting, and presentation of the results were frequently apparent. The poorly presented survival analyses emphasize the problems addressed in the recommendations by Altman and colleagues [58]. For example, to conduct the meta-analyses, we made 120 attempts to obtain estimates of the HR and its CI from the data/results provided, but only 79 of these proved successful. The remaining 41 were indirectly calculated using the raw individual patient data available or the survival curve plot. The HR and its CI (or loge(HR) and its variance) provide an important estimate of the difference in the risk of death (for OS) or disease recurrence/death (for DFS) between two groups of patients, but this is often given only as an inexact p value.

The indirect methods suggested by Parmar and colleagues [19] were found to be particularly crucial. To maximize the raw data mining, 18 arguments (see materials and methods for the details) in the article were extracted to indirectly calculate the lnHR and varlnHR. In some articles, the authors did not report the individual personal data (IPD) or the 18 arguments. However, the survival curve plot(s) were illustrated, and an R package was developed to extract the data to indirectly obtain the lnHR and varlnHR. This approach represents an innovative extension of the 11 methods summarized by Riley and co-workers [20] (Appendix 3).

### Clinical validities of CEA and CA19-9

We specifically investigated the clinical practices of the top two most studied markers, CEA and CA19-9, which are both diagnostic and prognostic markers for CRC and have significant heterogeneity and asymmetry. For CEA, a lack of sensitivity and specificity, when combined with the low prevalence of CRC in asymptomatic populations, preclude the use of CEA in screening for CRC [59]–[61]. In agreement with American Society of Clinical Oncology (ASCO) [62], [63] and European Group on Tumor Markers (EGTM) recommendations [64], [65], the National Academy of Clinical Biochemistry (NACB) Panel states that CEA cannot be used in diagnosis healthy subjects for early CRC. The patient stage at initial diagnosis is universally used to determine prognosis in patients with CRC. Several studies, however, have demonstrated that preoperative concentrations of CEA can also provide prognostic information which, in some situations, has been found to be independent of stage [59]–[61], [66]. Indeed, in some studies, CEA was found to be prognostic in patients with Stage II disease [59]–[61]. Preoperative concentrations of CEA might thus be combined with other factors to identify those Stage II colonic cancer patients who are candidates for adjuvant chemotherapy. There is, however, no evidence at present for a beneficial effect of adjuvant chemotherapy in either Stage II patients, as a whole, or in those with Stage II disease and high preoperative serum CEA concentrations. In agreement with other expert panels [62]–[65], the NACB Panel states that preoperative CEA levels should be measured in newly diagnosed CRC patients. CEA levels may be combined with histopathological parameters to determine which patients with Stage II colon cancer should receive adjuvant chemotherapy. However, as mentioned above, there is currently no evidence that Stage II colon cancer patients with elevated concentrations benefit from adjuvant chemotherapy. The CA 19-9 assay detects a mucin containing the sialated Lewis-a pentasaccharide epitope, fucopentaose II [67]. CA 19-9 is a less sensitive marker than CEA for CRC [68], [69]. Preliminary findings suggest that like CEA, preoperative concentrations of CA 19-9 are also prognostic in patients with CRC [70]–[75]. Based on available data, routine measurement of CA 19-9 as both diagnostic and prognostic markers cannot be recommended by either the ASCO [76] or EGTM [77] for patients with CRC.

## Conclusions

Our systematic review summarizes the evidence about the accuracy of serum diagnostic and prognostic tests for colorectal cancer (CRC). However, the majority of these markers have only been reported in a single study (diagnostic markers: 73 in 92, 79.3%; prognostic markers: 23 in 44, 52%). The cut-offs of those markers with more than three repetition studies present apparent fluctuations, and the effect sizes of the same marker in different studies generally demonstrate significant heterogeneity. The quality of studies addressing the diagnostic and prognostic accuracy of tests was poor, and the results were highly heterogeneous. Thus, like many reviewers of such studies, the present authors do not feel that the existing literature is strong enough to form a basis for clinical decisions, but the current systematic review can, we believe, highlight underlying problems on CRC serum markers and improve studies on CRC markers in the future, for example, exploring novel marker or constructing a “combination” marker composed of a few high-weights markers to arrive at clinically useful requirements.

## Supporting Information

Table S1 **List of serum or plasma markers in CRC that were identified by the systematic review together with the number of papers overall and within each clinical area.**
(XLS)

Table S2 **Study characteristics and quality of the included studies.** See Whiting et al. [17] for criteria on quality assessment. Items were scored 1 = yes, 2 = no, 3 = unclear. The reference IDs in the 2nd column are prefaced by a ‘D’ and listed in Appendix 4 in Materials S1
(XLS)

Table S3 **The complete summary of the performance of all serum diagnostic markers for colorectal cancer.** Note: reference IDs to these studies are prefaced by a ‘D’ and listed in Appendix 4 in Materials S1. In the Data acquisition(direct/indirect) column, ‘d’ means that the four core values, True Positive (TP), False Positive (FP), True Negative (TN) and False Negative (FN), of one diagnostic marker can be directly extracted from the study; otherwise, ‘i’ means the four values can indirectly extracted from the study and extrapolated from other relevant values. N/A means the value is not available. OR = (TP/FP)/(TN/FN), varlnOR = 1/TP+1/FP+1/TN+1/FN.
(XLS)

Table S4 **Study characteristics and quality of included prognosis papers.** Notes: An assessment of study methodology was performed according to REMARK study design [18], which includes 20 items. For any criterion not fulfilled according to the REMARK requirement, one point was deducted from a maximum of 20. Two independent investigators were assessed the eligibility criteria and quality scoring. Any disagreement was resolved by discussion. The scores of these studies ranged between 16 and 19.
(XLS)

Table S5 **Studies investigating the prognostic serum markers of colorectal cancer.** Note: The reference IDs to these studies are prefaced by a ‘P’ and listed in Appendix 5 in Materials S1. No of Patients (+) or (−) means the number of patients with positive or negative serological test results, defined by the level of the colorectal cancer marker. OS: overall survival; DFS: disease-free survival. In the Data acquisition (direct/indirect) column, ‘d’ means that the three core values, Hazard Ratio (HR), Lower Limit (LL), and Upper Limit (UL), of one prognostic marker can be directly extracted from the study; otherwise, ‘i’ means that the four values can be indirectly extracted from the study and extrapolated from other relevant values.
(XLS)

Checklist S1 **(PRISMA)**
(DOC)

Materials S1 **Supporting Information.**
(DOC)
