# Supplementary material for: A Systematic Review and Meta-Analysis of Diagnostic and Prognostic Serum Biomarkers of Colorectal Cancer
Source: PLoS One. 2014 Aug 8;9(8):e103910. doi: 10.1371/journal.pone.0103910 (PMC4126674; doi:10.1371/journal.pone.0103910)
Supplement: Materials S1 — Supporting Information. (DOC) [file pone.0103910.s007.doc]

# Supplementary Materials (Online only)

# Appendix 1

The keyword and the “associated” words for literature search of colorectal cancer

| Key words | “associated” words |
| --- | --- |
| Colorectal | CRC (colorectal cancer), colorectal, large intestine and large bowel |
| Colon | colon and colonic |
| rectal | rectal and rectum |
| cancer | cancer, carcinoma, tumor, malignancy and neoplasma |
| serum | serum, sera, plasma, blood |
| marker | marker(s), biomarker(s), signature molecule, molecular marker(s), marker(s) biological, biological marker(s), biologic markers and mark |

# Appendix 2

**The search strategy**

Search syntax in Pubmed and Embase: (colorectal OR large intestine OR large bowel OR colon OR colonic OR rectal OR rectum) AND (cancer OR carcinoma OR tumor OR neoplasm OR cancers) AND (serum OR sera OR serums OR blood OR plasma) AND (marker OR signature molecule OR molecular marker OR markers OR biomarkers OR biomarker OR mark)

| Database | Search strategy in details |
| --- | --- |
| pubmed | Colorectal cancer {Including Limited Related Terms}  (colorectal[Title/Abstract] OR (large[Title/Abstract] AND intestine[Title/Abstract]) OR (large[Title/Abstract] AND bowel[Title/Abstract]) OR colon[Title/Abstract] OR colonic[Title/Abstract] OR rectal[Title/Abstract] OR rectum[Title/Abstract]) AND (cancer[Title/Abstract] OR carcinoma[Title/Abstract] OR tumor[Title/Abstract] OR neoplasm[Title/Abstract] OR cancers[Title/Abstract]) AND (serum[Title/Abstract] OR sera[Title/Abstract] OR serums[Title/Abstract] OR blood[Title/Abstract] OR plasma[Title/Abstract]) AND (marker[Title/Abstract] OR (signature[Title/Abstract] AND molecule[Title/Abstract]) OR (molecular[Title/Abstract] AND marker[Title/Abstract]) OR markers[Title/Abstract] OR biomarkers[Title/Abstract] OR biomarker[Title/Abstract] OR mark[Title/Abstract]) AND ("humans"[MeSH Terms] AND (Clinical Trial[ptyp] OR Editorial[ptyp] OR Letter[ptyp] OR Case Reports[ptyp] OR Classical Article[ptyp] OR Clinical Conference[ptyp] OR Clinical Trial, Phase I[ptyp] OR Clinical Trial, Phase II[ptyp] OR Clinical Trial, Phase III[ptyp] OR Clinical Trial, Phase IV[ptyp] OR Controlled Clinical Trial[ptyp] OR Corrected and Republished Article[ptyp] OR English Abstract[ptyp] OR Journal Article[ptyp] OR Multicenter Study[ptyp]) AND English[lang] AND (cancer[sb] OR medline[sb])) |
| Embase | colorectal OR large AND 'intestine'/syn OR large AND 'bowel'/syn OR 'colon'/syn OR colonic OR 'rectal'/syn OR 'rectum'/syn AND ('cancer'/syn OR 'carcinoma'/syn OR 'tumor'/syn OR neoplasma OR 'cancers'/syn) AND ('serum'/syn OR sera OR serums OR 'blood'/syn OR 'plasma'/syn) AND ('marker'/syn OR signature AND 'molecule'/syn OR molecular AND 'marker'/syn OR markers OR biomarkers OR 'biomarker'/syn OR mark) AND ([controlled clinical trial]/lim OR [randomized controlled trial]/lim) AND ([article]/lim OR [article in press]/lim OR [editorial]/lim OR [letter]/lim) AND ([biochemistry]/lim OR [cancer]/lim OR [genetics]/lim OR [internal medicine]/lim OR [public health]/lim) AND [humans]/lim AND [english]/lim AND [1-1-1950]/sd NOT [18-10-2011]/sd |

# Appendix 3

**Statistical methods used to obtain estimates of log_e_(HR) and its variance**

The following describes the methods that were used to obtain estimates of log_e_(HR) and its variance (var[loge(HR)]) from all the occasions desired. The methods are based on those of Parmar and co-workers [1].

1. Given loge(HR) and var(HR):

Extract these direct estimates

1. Given HR and var(HR):

Calculate log_e_(HR)

Calculate a 95% CI for HR = HR ± 1.96 × SE(HR)

Calculate a 95% CI for log_e_(HR) = loge (95% HR CI)

Then use (4)

1. Given HR and an αi%CI:

Use logs to obtain log_e_(HR) and its 95% CI

Then use (4)

1. Given log_e_(HR) and an αi% CI:

Calculate

$$\mathrm{var}[\log_{e}(HR)]=(\frac{upper CI_{i}-lower CI_{i}}{2\Phi^{-1}(1-\alpha_{i}/2)})^{2}$$

1. Given HR and a *p* value (*p_i_*):

Calculate log_e_(HR) and use (6)

1. Given loge(HR) and a p value:

Calculate

$$\mathrm{var}[\log_{e}(HR)]\approx(\frac{\log_{e}(HR)}{\Phi^{-1}(1-\alpha_{i}/2)})^{2}$$

1. Given a *p* value (*p_i_*) for log_e_(HR) or HR, and the total number of deaths/recurrences (*O_i_*) and the group sizes are unequal with sizes n_1_and n_2_:

Calculate

$$\begin{matrix} \log_{e}(HR)\approx\frac{[\sqrt{O_{i}n_{1i}n_{2i}}/(n_{1i}+n_{2i})]\Phi^{-1}(1-p_{i}/2)}{O_{i}n_{1i}n_{2i}/(n_{1i}+n_{2i})^{2}} \\ \mathrm{var}[\log_{e}(HR)]\approx\frac{(n_{1i}+n_{2i})^{2}}{O_{i}n_{1i}n_{2i}} \end{matrix}$$

1. Given the χ2 statistic from the log-rank/Mantel–Haenszel test or Cox regression or Wilcoxon test comparing two groups of patients defined by marker status and the total number of deaths/recurrences (*O_i_*) in each group:

Use (7), since

$$\sqrt{\log-rank statistic}=\Phi^{-1}(1-\frac{p_{i}}{2})$$

1. Given individual patient data (IPD) that include initial mark value, follow-up time and final known status:

Calculate the direct estimate by using a Cox proportional hazards model

1. Given a survival curve with censoring points on it:

Estimate the observed number of events and patients at risk from each group at each event time, and use these to estimate the expected number of events for each group.

Then

$$\log_{e}(HR)=\frac{O_{1}/E_{1}}{O_{2}/E_{2}} , \mathrm{var}[\log_{e}(HR)]=\frac{1}{E_{1}}+\frac{1}{E_{2}}$$

1. Given an HR and only group numbers and group events:

Calculate log_e_(HR)

Calculate var[log_e_(HR)] from (7)

**Reference:**

1. Parmar MKB, Torri V, Stewart L. Extracting summary statistics to perform meta-analyses of the published literature for survival endpoints. Stat Med 1998;17:2815–34.

# Appendix 4

The references to these studies are prefaced by a ‘D’.

D1. Wild N, Andres H, Rollinger W, et al. A combination of serum markers for the early detection of colorectal cancer. Clinical cancer research : an official journal of the American Association for Cancer Research. 2010;16(24):6111-6121.

D2. Kovacevic D, Sonicki Z, Kusic Z, et al. Preoperative serum levels of c-erbB-2 do not seem to be useful in management of patients with rectal cancer. International journal of colorectal disease. 2007;22(7):827-831.

D3. Herszenyi L, Farinati F, Cardin R, et al. Tumor marker utility and prognostic relevance of cathepsin B, cathepsin L, urokinase-type plasminogen activator, plasminogen activator inhibitor type-1, CEA and CA 19-9 in colorectal cancer. BMC cancer. 2008;8:194.

D4. Lee H, Rhee H, Kang HJ, et al. Macrophage migration inhibitory factor may be used as an early diagnostic marker in colorectal carcinomas. American journal of clinical pathology. 2008;129(5):772-779.

D5. Fernandes LC, Kim SB & Matos D Cytokeratins and carcinoembryonic antigen in diagnosis, staging and prognosis of colorectal adenocarcinoma. World journal of gastroenterology : WJG. 2005;11(5):645-648.

D6. Saito N & Kameoka S Serum laminin is an independent prognostic factor in colorectal cancer. International journal of colorectal disease. 2005;20(3):238-244.

D7. Nakajima Identification of Cystatin SN as a novel tumor marker for colorectal cancer. International Journal of Oncology. 2009;35(01).

D8. Coban S, Ozkan H, Koklu S, et al. The utility of serum receptor-binding cancer antigen expressed on SiSo cells in gastrointestinal tract cancers. Canadian journal of gastroenterology = Journal canadien de gastroenterologie. 2006;20(9):593-596.

D9. Guadagni F, Roselli M, Cosimelli M, et al. TAG-72 (CA 72-4 assay) as a complementary serum tumor antigen to carcinoembryonic antigen in monitoring patients with colorectal cancer. Cancer. 1993;72(7):2098-2106.

D10. Marrelli D, Caruso S, Neri A, et al. Clinical utility of serum tumor markers in the diagnosis of malignant intestinal occlusion. A prospective observational study. The International Journal of Biological Markers. 2011;26(1):58-64.

D11. Guadagni F, Roselli M, Amato T, et al. Clinical evaluation of serum tumor-associated glycoprotein-72 as a novel tumor marker for colorectal cancer patients. Journal of surgical oncology. Supplement. 1991;2:16-20.

D12. Guadagni F, Roselli M, Cosimelli M, et al. Biologic evaluation of tumor-associated glycoprotein-72 and carcinoembryonic antigen expression in colorectal cancer, Part I. Diseases of the colon and rectum. 1994;37(2 Suppl):S16-23.

D13. van Kamp GJ, von Mensdorff-Pouilly S, Kenemans P, et al. Evaluation of colorectal cancer-associated mucin CA M43 assay in serum. Clinical chemistry. 1993;39(6):1029-1032.

D14. Wei JS, Chung NC, Wei LL, et al. High-molecular-mass alkaline phosphatase as a tumor marker for colorectal cancer: comparison of two test methods. Clinical chemistry. 1993;39(3):540-543.

D15. Severini G Glutathione S-transferase activity in patients with cancer of the digestive tract. Journal of cancer research and clinical oncology. 1993;120(1-2):112-114.

D16. Kuusela P, Haglund C & Roberts PJ Comparison of a new tumour marker CA 242 with CA 19-9, CA 50 and carcinoembryonic antigen (CEA) in digestive tract diseases. British journal of cancer. 1991;63(4):636-640.

D17. Kuusela P, Haglund C, Roberts PJ, et al. Comparison of CA-50, a new tumour marker, with carcinoembryonic antigen (CEA) and alpha-fetoprotein (AFP) in patients with gastrointestinal diseases. British journal of cancer. 1987;55(6):673-676.

D18. Heptner G, Domschke S & Domschke W Comparison of CA 72-4 with CA 19-9 and carcinoembryonic antigen in the serodiagnostics of gastrointestinal malignancies. Scandinavian journal of gastroenterology. 1989;24(6):745-750.

D19. Baumann M, Brand K, Giedl J, et al. Significance of serum phosphohexose isomerase in gastrointestinal cancer at different stages. Oncology. 1988;45(3):153-158.

D20. Haglund C, Kuusela P, Roberts P, et al. Tumour marker CA 125 in patients with digestive tract malignancies. Scandinavian journal of clinical and laboratory investigation. 1991;51(3):265-270.

D21. Renehan AG, Jones J, Potten CS, et al. Elevated serum insulin-like growth factor (IGF)-II and IGF binding protein-2 in patients with colorectal cancer. British journal of cancer. 2000;83(10):1344-1350.

D22. Dbouk HA, Tawil A, Nasr F, et al. Significance of CEA and VEGF as Diagnostic Markers of Colorectal Cancer in Lebanese Patients. The open clinical cancer journal. 2007;1:1-5.

D23. Chen JS, Chen KT, Fan WC, et al. Combined analysis of survivin autoantibody and carcinoembryonic antigen biomarkers for improved detection of colorectal cancer. Clinical chemistry and laboratory medicine : CCLM / FESCC. 2010;48(5):719-725.

D24. Pasanen P, Eskelinen M, Kulju A, et al. Tumour-associated trypsin inhibitor (TATI) in patients with colorectal cancer: a comparison with CEA, CA 50 and CA 242. Scandinavian journal of clinical and laboratory investigation. 1995;55(2):119-124.

D25. Takeda A, Otani Y, Iseki H, et al. Clinical significance of large tenascin-C spliced variant as a potential biomarker for colorectal cancer. World journal of surgery. 2007;31(2):388-394.

D26. Broll R, Erdmann H, Duchrow M, et al. Vascular endothelial growth factor (VEGF)--a valuable serum tumour marker in patients with colorectal cancer? European journal of surgical oncology : the journal of the European Society of Surgical Oncology and the British Association of Surgical Oncology. 2001;27(1):37-42.

D27. Okuyama N, Ide Y, Nakano M, et al. Fucosylated haptoglobin is a novel marker for pancreatic cancer: a detailed analysis of the oligosaccharide structure and a possible mechanism for fucosylation. International journal of cancer. Journal international du cancer. 2006;118(11):2803-2808.

D28. Otani T, Iwasaki M, Sasazuki S, et al. Plasma C-reactive protein and risk of colorectal cancer in a nested case-control study: Japan Public Health Center-based prospective study. Cancer epidemiology, biomarkers & prevention : a publication of the American Association for Cancer Research, cosponsored by the American Society of Preventive Oncology. 2006;15(4):690-695.

D29. Erlinger TP, Platz EA, Rifai N, et al. C-reactive protein and the risk of incident colorectal cancer. JAMA : the journal of the American Medical Association. 2004;291(5):585-590.

D30. Ito Y, Suzuki K, Tamakoshi K, et al. Colorectal cancer and serum C-reactive protein levels: a case-control study nested in the JACC Study. Journal of epidemiology / Japan Epidemiological Association. 2005;15 Suppl 2:S185-189.

D31. Karayiannakis AJ, Syrigos KN, Zbar A, et al. Clinical significance of preoperative serum vascular endothelial growth factor levels in patients with colorectal cancer and the effect of tumor surgery. Surgery. 2002;131(5):548-555.

D32. De Vita F, Orditura M, Lieto E, et al. Elevated perioperative serum vascular endothelial growth factor levels in patients with colon carcinoma. Cancer. 2004;100(2):270-278.

D33. Paterson AC, Leeding KS, Bach LA, et al. More about: prospective study of colorectal cancer risk in men and plasma levels of insulin-like growth factor (IGF)-I and IGF-binding protein-3. Journal of the National Cancer Institute. 2000;92(23):1947-1950.

D34. Palmqvist R, Hallmans G, Rinaldi S, et al. Plasma insulin-like growth factor 1, insulin-like growth factor binding protein 3, and risk of colorectal cancer: a prospective study in northern Sweden. Gut. 2002;50(5):642-646.

D35. Giovannucci E, Pollak MN, Platz EA, et al. A prospective study of plasma insulin-like growth factor-1 and binding protein-3 and risk of colorectal neoplasia in women. Cancer epidemiology, biomarkers & prevention : a publication of the American Association for Cancer Research, cosponsored by the American Society of Preventive Oncology. 2000;9(4):345-349.

D36. Rinaldi S, Cleveland R, Norat T, et al. Serum levels of IGF-I, IGFBP-3 and colorectal cancer risk: results from the EPIC cohort, plus a meta-analysis of prospective studies. International journal of cancer. Journal international du cancer. 2010;126(7):1702-1715.

D37. Motoo Y, Satomura Y, Kawakami H, et al. Serum levels of tumor-associated glycoprotein (TAG-72) in digestive cancers. Oncology. 1990;47(6):456-462.

D38. Motoo Y, Sawabu N, Yamaguchi Y, et al. Serum levels of c-erbB-2 protein in digestive diseases. Journal of gastroenterology. 1994;29(5):616-620.

D39. Narai S, Watanabe M, Hasegawa H, et al. Significance of transforming growth factor beta1 as a new tumor marker for colorectal cancer. International journal of cancer. Journal international du cancer. 2002;97(4):508-511.

D40. Jelski W, Mroczko B & Szmitkowski M The diagnostic value of alcohol dehydrogenase (ADH) isoenzymes and aldehyde dehydrogenase (ALDH) measurement in the sera of colorectal cancer patients. Digestive diseases and sciences. 2010;55(10):2953-2957.

D41. Wilson S, Wakelam MJ, Hobbs RF, et al. Evaluation of the accuracy of serum MMP-9 as a test for colorectal cancer in a primary care population. BMC cancer. 2006;6:258.

D42. Fentz AK, Sporl M, Spangenberg J, et al. Detection of colorectal adenoma and cancer based on transthyretin and C3a-desArg serum levels. Proteomics. Clinical applications. 2007;1(6):536-544.

D43. Leman ES, Schoen RE, Magheli A, et al. Evaluation of colon cancer-specific antigen 2 as a potential serum marker for colorectal cancer. Clinical cancer research : an official journal of the American Association for Cancer Research. 2008;14(5):1349-1354.

D44. Leman ES, Schoen RE, Weissfeld JL, et al. Initial analyses of colon cancer-specific antigen (CCSA)-3 and CCSA-4 as colorectal cancer-associated serum markers. Cancer research. 2007;67(12):5600-5605.

D45. Chester KA & Begent RH Circulating immune complexes (CIC), carcinoembryonic antigen (CEA) and CIC containing CEA as markers for colorectal cancer. Clinical and experimental immunology. 1984;58(3):685-693.

D46. Kozwich DL, Kramer LC, Mielicki WP, et al. Application of cancer procoagulant as an early detection tumor marker. Cancer. 1994;74(4):1367-1376.

D47. Ma J, Giovannucci E, Pollak M, et al. A Prospective Study of Plasma C-Peptide and Colorectal Cancer Risk in Men. JNCI Journal of the National Cancer Institute. 2004;96(7):546-553.

D48. Flamini E, Mercatali L, Nanni O, et al. Free DNA and carcinoembryonic antigen serum levels: an important combination for diagnosis of colorectal cancer. Clinical cancer research : an official journal of the American Association for Cancer Research. 2006;12(23):6985-6988.

D49. Duraker N, Can D & Parilti M Measurement of serum total and free prostate-specific antigen in women with colorectal carcinoma. British journal of cancer. 2002;86(2):203-206.

D50. Toiyama Y, Miki C, Inoue Y, et al. Soluble intercellular adhesion molecule-1 as a prognostic marker for stage II colorectal cancer patients. Annals of surgical oncology. 2008;15(6):1617-1624.

D51. Severini G, Diana L, Di Giovannandrea R, et al. A study of serum glycosidases in cancer. Journal of cancer research and clinical oncology. 1995;121(1):61-63.

D52. Arai M, Sakamoto K, Otsuka H, et al. Detection of tumor associated antigen, PA8-15, in sera from pancreatic and gastrointestinal carcinoma patients. Japanese journal of clinical oncology. 1990;20(2):145-153.

D53. Pucci S, Bonanno E, Sesti F, et al. Clusterin in stool: a new biomarker for colon cancer screening? The American journal of gastroenterology. 2009;104(11):2807-2815.

D54. Pinczower GD, Gianello RD, Williams RP, et al. Monoclonal antibody 4D3 detects small intestinal mucin antigen (SIMA)--glycoprotein in the serum of patients with colorectal cancer. International journal of cancer. Journal international du cancer. 1993;54(3):391-396.

D55. Shim KS, Kim KH, Park BW, et al. Increased serum levels of transforming growth factor-alpha in patients with colorectal cancer. Diseases of the colon and rectum. 1998;41(2):219-224.

D56. Shim KS, Kim KH, Han WS, et al. Elevated serum levels of transforming growth factor-beta1 in patients with colorectal carcinoma: its association with tumor progression and its significant decrease after curative surgical resection. Cancer. 1999;85(3):554-561.

D57. Kornek G, Depisch D, Temsch EM, et al. Comparative analysis of cancer-associated antigen CA-195, CA 19-9 and carcinoembryonic antigen in diagnosis, follow-up and monitoring of response to chemotherapy in patients with gastrointestinal cancer. J Cancer Res Clin Oncol. 1991;117:493 – 496.

D58. Kuusela P, Haglund C, Roberts PJ. Comparison of a new tumour marker CA242 with CA19-9, CA50 and carcinoembryonic antigen (CEA) in digestive tract diseases. Br J Cancer. 1991;63:636 – 640.

D59. Nilsson O, Johansson C, Glimelius B, et al. Sensitivity and specificity of CA242 in gastro-intestinal cancer. A comparison with CEA, CA50 and CA 19-9. Br J Cancer. 1992;65:215 – 221.

D60. Huber K, Kirchheimer JC, Sedlmayer A, et al. Clinical value of determination of urokinase-type plasminogen activator antigen in plasma for detection of colorectal cancer:comparison with circulating tumor-associated antigens CA19-9 and carcinoembryonic antigen. Cancer Res. 1993;53:1788 –1793.

D61. Carpelan-Holmstrom M, Haglund C, Kuusela P, et al. Preoperative serum levels of CEA and CA 242 in colorectal cancer. Br J Cancer. 1995;71:868 – 872.

D62. Fernandez-Fernandez L, Tejero E, Tieso A. Significance of CA 72-4 in colorectal carcinoma.Comparison with CEA and CA 19-9. Eur J Surg Oncol. 1995;21:388 – 390.

D63. Carpelan-Holmstrom M, Louhimo J, Stenman UH, Alfthan H, Jarvinen H, Haglund C. Estimating the probability of cancer with several tumor markers in patients with colorectal disease. Oncology. 2004;66:296 – 302.

D64. Blake KE, Dalbow MH, Concannon JP, et al. Clinical significance of the preoperative plasma carcinoembryonic antigen (CEA) level in patients with carcinoma of the large bowel. Dis Colon Rectum. 1982; 25:24 – 32.

D65. Kuusela P, Jalanko H, Roberts P, et al. Comparison of CA 19-9 and carcinoembryonic antigen (CEA) levels in the serum of patients with colorectal diseases. Br J Cancer 1984;49:135 –139.

D66. Wang FM, Tsai LC, Chang ZN,et al. The significance of CA19-9 tumor antigen in the serum of patients with carcinomas. Proc Natl Sci Counc Repub China B. 1985;9:119 – 125.

D67. Eskelinen M, Pasanen P, Kulju A, et al. Clinical evaluation of serum tumour markers CEA, CA 50 and CA 242 in colorectal cancer. Anticancer Res 1994;14:1427 – 1432.

D68. Paganuzzi M, Onetto M, de Paoli M, et al. Carcinoembryonic antigen (CEA) in serum and bile of colorectal cancer patients with or without detectable liver metastases. Anticancer Res 1994;14:1409 – 1412.

D69. von Kleist S. Comparative evaluation of four tumor markers, CA 242, Ca 19/9, TPA, and CEA in carcinomas of the colon. Anticancer Res. 1996;16:2325 –2332.

D70. Spila A, Ferroni P, Cosimelli M, et al. Comparative analysis of CA 242 and CA 19-9 serum tumor markers in colorectal cancer patients. A longitudinal evaluation. Anticancer Res. 2001;21:1263 – 1270.

D71. Carpelan-Holmstrom M, Louhimo J, Stenman UH, et al. CEA, CA 19-9 and CA 72-4 improve the diagnostic accuracy in gastrointestinal cancers. Anticancer Res. 2002;22: 2311 – 2316.

D72. Castaldi F, Marino M, Beneduce L, et al. Detection of circulating CEA-IgM complexes in early stage colorectal cancer. Int J Biol Markers. 2005;20:204 –208.

D73. Thomas WM, Robertson JF, Price MR, et al. Failure of CA19-9 to detect asymptomatic colorectal carcinoma. Br J Cancer. 1991;63:975 – 976.

D74. Spila A, Ferroni P, Cosimelli M, et al. Evaluation of the CA 242 tumor antigen as a potential serum marker for colorectal cancer. Anticancer Res. 1999;19:1363 – 1368.

D75. Hyodo I, Doi T, Endo H, et al. Clinical significance of plasma vascular endothelial growth factor in gastrointestinal cancer. Eur J Cancer 1998;34:2041 – 2045.

D76. Kumar H, Heer K, Lee PW, et al. Preoperative serum vascular endothelial growth factor can predict stage in colorectal cancer. Clin Cancer Res. 1998;4:1279 – 1285.

D77. Tsai WS, Changchien CR, Yeh CY, et al. Preoperative plasma vascular endothelial growth factor but not nitrite is a useful complementary tumor marker in patients with colorectal cancer. Dis Colon Rectum. 2006;49:883 – 894.

D78. Holmgren J, Lindholm L, Persson B, et al. Detection by monoclonal antibody of carbohydrate antigen CA 50 in serum of patients with carcinoma. Br Med J (Clin Res Ed). 1984;288:1479 – 1482.

D79. Pasanen P, Eskelinen M, Kulju A, et al. Tumour-associated trypsin inhibitor (TATI) in patients with colorectal cancer: a comparison with CEA, CA 50 and CA 242. Scand J Clin Lab Invest. 1995;55:119 – 24.

D80. Holten-Andersen MN, Christensen IJ, Nielsen HJ, et al. Total levels of tissue inhibitor of metalloproteinases 1 in plasma yield high diagnostic sensitivity and specificity in patients with colon cancer. Clin Cancer Res. 2002;8:156 – 164.

D81. Hammel P, Boissier B, Chaumette MT, et al. Detection and monitoring of serum p53 antibodies in patients with colorectal cancer. Gut. 1997;40:356 – 361.

D82. Broll R, Duchrow M, Oevermann E, et al. p53 autoantibodies in sera of patients with a colorectal cancer and their association to p53 protein concentration and p53 immunohistochemistry in tumor tissue. Int J Colorectal Dis. 2001;16:22 – 27.

D83. Chang SC, Lin JK, Lin TC, Liang WY. Genetic alteration of p53, but not overexpression of intratumoral p53 protein, or serum p53 antibody is a prognostic factor in sporadic colorectal adenocarcinoma. Int J Oncol. 2005;26:65 – 75.

D84. Zhang B, Chen JY, Chen DD, et al. Tumor type M2 pyruvate kinase expression in gastric cancer, colorectal cancer and controls. World J Gastroenterol. 2004;10:1643 –1646.

D85. Schneider J, Bitterlich N, Schulze G. Improved sensitivity in the diagnosis of gastro-intestinal tumors by fuzzy logic-based tumor marker profiles including the tumor M2-PK. Anticancer Res. 2005;25:1507 – 1515.

D86. Yedema KA, Kenemans P, Wobbes T, et al. Carcinoma-associated mucin serum markers CA M26 and CA M29: efficacy in detecting and monitoring patients with cancer of the breast, colon, ovary, endometrium and cervix. Int J Cancer. 1991;47:170 – 179.

D87. van Kamp GJ, von Mensdorff-Pouilly S, Kenemans P, et al. Evaluation of colorectal cancer-associated mucin CA M43 assay in serum. Clin Chem. 1993;39:1029 – 1032.

D88. Yamaguchi A, Kurosaka Y, Ishida T, et al. Clinical significance of tumor marker NCC-ST 439 in large bowel cancers. Dis Colon Rectum 1991;34:921 – 924.

D89. Xia Q, Kong XT, Zhang GA, et al. Proteomics-based identification of DEAD-box protein 48 as a novel autoantigen, a prospective serum marker for pancreatic cancer. Biochem Biophys Res Commun. 2005;330:526 – 532.

D90. Reipert BM, Tanneberger S, Pannetta A, et al. Increase in autoantibodies against Fas (CD95) during carcinogenesis in the human colon: a hope for the immunoprevention of cancer? Cancer Immunol Immunother. 2005;54:1038 –1042.

D91. Mroczko B, Szmitkowski M, Wereszczynska-Siemiatkowska U, Okulczyk B. Stem cell factor (SCF) and interleukin 3 (IL-3) in the sera of patients with colorectal cancer. Dig Dis Sci. 2005;50:1019 – 1024.

D92. Dudouet B, Jacob L, Beuzeboc P, et al. Presence of villin, a tissuespecific cytoskeletal protein, in sera of patients and an initial clinical evaluation of its value for the diagnosis and follow-up of colorectal cancers. Cancer Res. 1990;50:438 – 443.

D93. Riedl S, Bodenmuller H, Hinz U, et al. Significance of tenascin serum level as tumor marker in primary colorectal carcinoma. Int J Cancer. 1995;64:65 – 69.

D94. Ayude D, Fernandez-Rodriguez J, Rodriguez-Berrocal FJ, et al. Value of the serum a-L-fucosidase activity in the diagnosis of colorectal cancer. Oncology. 2000;59:310 – 316.

D95. Ayude D, Paez de la Cadena M, Cordero OJ, et al. Clinical interest of the combined use of serum CD26 and a-L-fucosidase in the early diagnosis of colorectal cancer. Dis Markers. 2003;19:267 – 272.

D96. Cordero OJ, Ayude D, Nogueira M, et al. Preoperative serum CD26 levels: diagnostic efficiency and predictive value for colorectal cancer. Br J Cancer 2000;83: 1139 – 1146.

D97. Ferroni P, Roselli M, Martini F, et al. Prognostic value of soluble P-selectin levels in colorectal cancer. Int J Cancer 2004; 111:404 – 408.

D98. Kerber A, Trojan J, Herrlinger K, et al. The new DR-70 immunoassay detects cancer of the gastrointestinal tract: a validation study. Aliment Pharmacol Ther. 2004; 20:983 – 987.

D99. Soroush AR, Zadeh HM, Moemeni M, et al. Plasma prolactin in patients with colorectal cancer. BMC Cancer. 2004;4:97.

D100. Melle C, Ernst G, Schimmel B, et al. Discovery and identification of adefensins as low abundant, tumor-derived serum mark ers in colorectal cancer. Gastroenterology. 2005;129:66 – 73.

D101. Roessler M, Rollinger W, Palme S, et al. Identification of nicotinamide N-methyltransferase as a novel serum tumor marker for colorectal cancer. Clin Cancer Res. 2005;11:6550 – 6557.

D102. Wei JS, Chung NC, Wei LL, et al. High-molecular-mass alkaline phosphatase as a tumor marker for colorectal cancer: comparison of two test methods. Clin Chem 1993;39:540 – 543.

D103. Chen YD, Zheng S, Yu JK, Hu X. Artificial neural networks analysis of surface-enhanced laser desorption/ionization mass spectra of serum protein pattern distinguishes colorectal cancer from healthy population. Clin Cancer Res. 2004;10:8380 – 8385.

D104. Yu JK, Chen YD, Zheng S. An integrated approach to the detection of colorectal cancer utilizing proteomics and bioinformatics. World J Gastroenterol. 2004;10:3127 – 3131.

# Appendix 5

The references to these studies are prefaced by a ‘P’.

P1. Webb A, Scott-Mackie P, Cunningham D, et al. The prognostic value of CEA, beta HCG, AFP, CA125, CA19-9 and C-erb B-2, beta HCG immunohistochemistry in advanced colorectal cancer. Ann Oncol. 1995;6(6):581-587.

P2. Martinez-Fernandez A, Garcia-Albeniz X, Pineda E, et al. Serum matrilysin levels predict outcome in curatively resected colorectal cancer patients. Ann Surg Oncol. 2009;16(5):1412-1420.

P3. Gaber A, Nodin B, Hotakainen K, et al. Increased serum levels of tumour-associated trypsin inhibitor independently predict a poor prognosis in colorectal cancer patients. BMC Cancer. 2010;10:498.

P4. Lomholt AF, Christensen IJ, Hoyer-Hansen G, et al. Prognostic value of intact and cleaved forms of the urokinase plasminogen activator receptor in a retrospective study of 518 colorectal cancer patients. Acta Oncol. 2010;49(6):805-811.

P5. Zheng CX, Zhan WH, Zhao JZ, et al. The prognostic value of preoperative serum levels of CEA, CA19-9 and CA72-4 in patients with colorectal cancer. World J Gastroenterol. 2001;7(3):431-434.

P6. Ma CJ, Hsieh JS, Wang WM, et al. Multivariate analysis of prognostic determinants for colorectal cancer patients with high preoperative serum CEA levels: prognostic value of postoperative serum CEA levels. Kaohsiung J Med Sci. 2006;22(12):604-609.

P7. Chen CC, Yang SH, Lin JK, et al. Is it reasonable to add preoperative serum level of CEA and CA19-9 to staging for colorectal cancer? J Surg Res. 2005;124(2):169-174.

P8. Hogdall CK, Christensen IJ, Stephens RW, et al. Serum tetranectin is an independent prognostic marker in colorectal cancer and weakly correlated with plasma suPAR, plasma PAI-1 and serum CEA. APMIS. 2002;110(9):630-638.

P9. Oussoultzoglou E, Rosso E, Fuchshuber P, et al. Perioperative carcinoembryonic antigen measurements to predict curability after liver resection for colorectal metastases: a prospective study. Arch Surg. 2008;143(12):1150-1158; discussion 1158-1159.

P10. De Vita F, Orditura M, Lieto E, et al. Elevated perioperative serum vascular endothelial growth factor levels in patients with colon carcinoma. Cancer. 2004;100(2):270-278.

P11. Ferroni P, Roselli M, Martini F, et al. Prognostic value of soluble P-selectin levels in colorectal cancer. International Journal of Cancer. 2004;111(3):404-408.

P12. Galizia G, Orditura M, Romano C, et al. Prognostic significance of circulating IL-10 and IL-6 serum levels in colon cancer patients undergoing surgery. Clin Immunol. 2002;102(2):169-178.

P13. Birgisson H, Nielsen HJ, Christensen IJ, et al. Preoperative plasma TIMP-1 is an independent prognostic indicator in patients with primary colorectal cancer: a prospective validation study. Eur J Cancer. 2010;46(18):3323-3331.

P14. Maurel J, Nadal C, Garcia-Albeniz X, et al. Serum matrix metalloproteinase 7 levels identifies poor prognosis advanced colorectal cancer patients. Int J Cancer. 2007;121(5):1066-1071.

P15. Louhimo J, Carpelan-Holmstrom M, Alfthan H, et al. Serum HCG beta, CA 72-4 and CEA are independent prognostic factors in colorectal cancer. Int J Cancer. 2002;101(6):545-548.

P16. Huh JW, Oh BR, Kim HR, et al. Preoperative carcinoembryonic antigen level as an independent prognostic factor in potentially curative colon cancer. J Surg Oncol. 2010;101(5):396-400.

P17. Chin KF, Greenman J, Gardiner E, et al. Pre-operative serum vascular endothelial growth factor can select patients for adjuvant treatment after curative resection in colorectal cancer. Br J Cancer. 2000;83(11):1425-1431.

P18. Werther K, Sørensen S, Christensen IJ, et al. Circulating Vascular Endothelial Growth Factor Six Months after Primary Surgery as a Prognostic Marker in Patients with Colorectal Cancer. Acta Oncologica. 2003;42(8):837-845.

P19. Koike Y, Miki C, Okugawa Y, et al. Preoperative C-reactive protein as a prognostic and therapeutic marker for colorectal cancer. J Surg Oncol. 2008;98(7):540-544.

P20. Tahara K, Mimori K, Iinuma H, et al. Serum matrix-metalloproteinase-1 is a bona fide prognostic marker for colorectal cancer. Ann Surg Oncol. 2010;17(12):3362-3369.

P21. Paganuzzi M, Bobbio B, Marroni P, et al. Prognostic Role of Serum Sialyl Lewis<sup>x</sup> (CD15s) in Colorectal Cancer. Oncology. 2003;65(1):52-59.

P22. Okugawa Y, Miki C, Toiyama Y, et al. Serum level of soluble vascular cell adhesion molecule 1 is a valuable prognostic marker in colorectal carcinoma. Dis Colon Rectum. 2009;52(7):1330-1336.

P23. Takagawa R, Fujii S, Ohta M, et al. Preoperative serum carcinoembryonic antigen level as a predictive factor of recurrence after curative resection of colorectal cancer. Ann Surg Oncol. 2008;15(12):3433-3439.

P24. Wei SC, Liang JT, Tsao PN, et al. Preoperative serum placenta growth factor level is a prognostic biomarker in colorectal cancer. Dis Colon Rectum. 2009;52(9):1630-1636.

P25. Nasif WA, Lotfy M, El-Sayed IH, et al. Implications of CEA and p53 overexpression in the poor prognosis of colorectal cancer. Med Oncol. 2006;23(2):237-244.

P26. Filella X, Molina R, Grau JJ, et al. Prognostic value of CA 19.9 levels in colorectal cancer. Ann Surg. 1992;216(1):55-59.

P27. Toiyama Y, Miki C, Inoue Y, et al. Serum hepatocyte growth factor as a prognostic marker for stage II or III colorectal cancer patients. Int J Cancer. 2009;125(7):1657-1662.

P28. Toiyama Y, Miki C, Inoue Y, et al. Soluble Intercellular Adhesion Molecule-1 as a Prognostic Marker for Stage II Colorectal Cancer Patients. Annals of Surgical Oncology. 2008;15(6):1617-1624.

P29. Park IJ, Choi GS,andJun SH. Prognostic value of serum tumor antigen CA19-9 after curative resection of colorectal cancer. Anticancer Res. 2009;29(10):4303-4308.

P30. Herszenyi L, Farinati F, Cardin R, et al. Tumor marker utility and prognostic relevance of cathepsin B, cathepsin L, urokinase-type plasminogen activator, plasminogen activator inhibitor type-1, CEA and CA 19-9 in colorectal cancer. BMC Cancer. 2008;8:194.

P31. Alabi AA, Suppiah A, Madden LA, et al. Preoperative serum vascular endothelial growth factor-a is a marker for subsequent recurrence in colorectal cancer patients. Dis Colon Rectum. 2009;52(5):993-999.

P32. Volkova E, Willis JA, Wells JE, et al. Association of angiopoietin-2, C-reactive protein and markers of obesity and insulin resistance with survival outcome in colorectal cancer. Br J Cancer. 2011;104(1):51-59.

P33. Karayiannakis AJ, Syrigos KN, Zbar A, et al. Clinical significance of preoperative serum vascular endothelial growth factor levels in patients with colorectal cancer and the effect of tumor surgery. Surgery. 2002;131(5):548-555.

P34. Broll R, Erdmann H, Duchrow M, et al. Vascular endothelial growth factor (VEGF)--a valuable serum tumour marker in patients with colorectal cancer? Eur J Surg Oncol. 2001;27(1):37-42.

P35. Ytting H, Christensen IJ, Thiel S, et al. Serum mannan-binding lectin-associated serine protease 2 levels in colorectal cancer: relation to recurrence and mortality. Clin Cancer Res. 2005;11(4):1441-1446.

P36. Ytting H, Christensen IJ, Thiel S, et al. Pre- and postoperative levels in serum of mannan-binding lectin associated serine protease-2 -a prognostic marker in colorectal cancer. Hum Immunol. 2008;69(7):414-420.

P37. Crozier JE, McKee RF, McArdle CS, et al. Preoperative but not postoperative systemic inflammatory response correlates with survival in colorectal cancer. Br J Surg. 2007;94(8):1028-1032.

P38. Nielsen HJ, Christensen IJ, Sorensen S, et al. Preoperative plasma plasminogen activator inhibitor type-1 and serum C-reactive protein levels in patients with colorectal cancer. The RANX05 Colorectal Cancer Study Group. Ann Surg Oncol. 2000;7(8):617-623.

P39. Nozoe T, Mori E, Takahashi I, et al. Preoperative elevation of serum C-reactive protein as an independent prognostic indicator of colorectal carcinoma. Surg Today. 2008;38(7):597-602.

P40. Giaginis C, Nikiteas N, Margeli A, et al. Serum tissue inhibitor of metalloproteinase 1 and 2 (TIMP-1 and TIMP-2) levels in colorectal cancer patients: associations with clinicopathological variables and patient survival. Int J Biol Markers. 2009;24(4):245-252.

P41. Holten-Andersen MN, Nielsen HJ, Sorensen S, et al. Tissue inhibitor of metalloproteinases-1 in the postoperative monitoring of colorectal cancer. Eur J Cancer. 2006;42(12):1889-1896.

P42. Cintin C, Johansen JS, Christensen IJ, et al. Serum YKL-40 and colorectal cancer. Br J Cancer. 1999;79(9-10):1494-1499.

P43. Cintin C, Johansen JS, Christensen IJ, et al. High serum YKL-40 level after surgery for colorectal carcinoma is related to short survival. Cancer. 2002;95(2):267-274.

P44. Brown DA, Ward RL, Buckhaults P, et al. MIC-1 serum level and genotype: associations with progress and prognosis of colorectal carcinoma. Clin Cancer Res. 2003;9(7):2642-2650.

P45. Kocsis J, Madaras B, Toth EK, et al. Serum level of soluble 70-kD heat shock protein is associated with high mortality in patients with colorectal cancer without distant metastasis. Cell Stress Chaperones. 2010;15(2):143-151.

P46. Carpelan-Holmstrom M, Haglund C, Lundin J, et al. Pre-operative serum levels of CA 242 and CEA predict outcome in colorectal cancer. Eur J Cancer. 1996;32A(7):1156-1161.

P47. Toiyama Y, Miki C, Inoue Y, et al. Serum immunosuppressive acidic protein reflects systemic deterioration of colorectal cancer patient condition. J Surg Oncol. 2008;97(5):404-408.

P48. Saito N,andKameoka S. Serum laminin is an independent prognostic factor in colorectal cancer. Int J Colorectal Dis. 2005;20(3):238-244.

P49. Ausch C, Buxhofer-Ausch V, Olszewski U, et al. Caspase-cleaved cytokeratin 18 fragment (M30) as marker of postoperative residual tumor load in colon cancer patients. Eur J Surg Oncol. 2009;35(11):1164-1168.

# Appendix 6

Diagnostic Figure 1S:


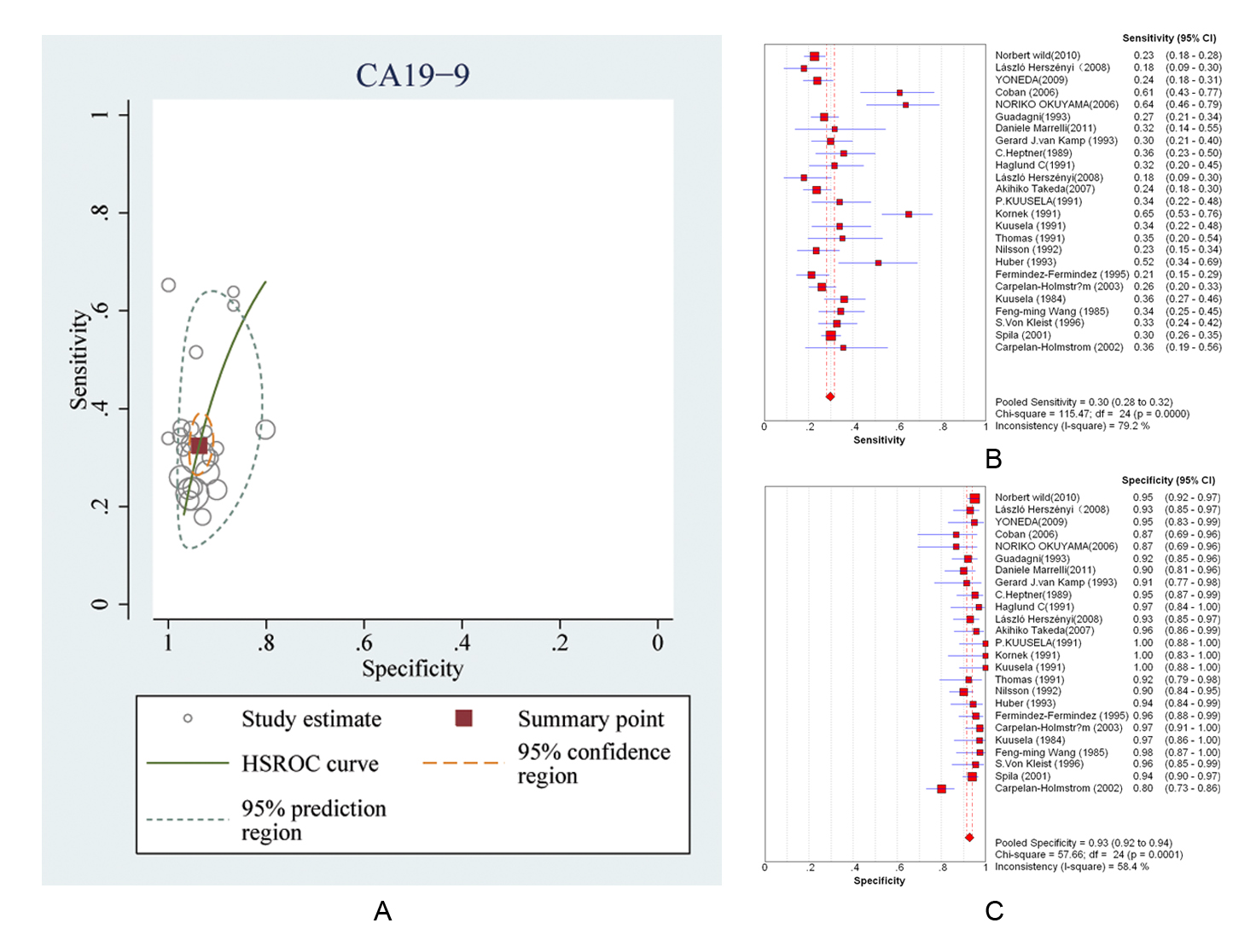
HSROC plot (A), forest plots of specificity (B) and sensitivity (C) of diagnostic marker CA19-9 for colorectal cancer

Diagnostic Figure 2S:


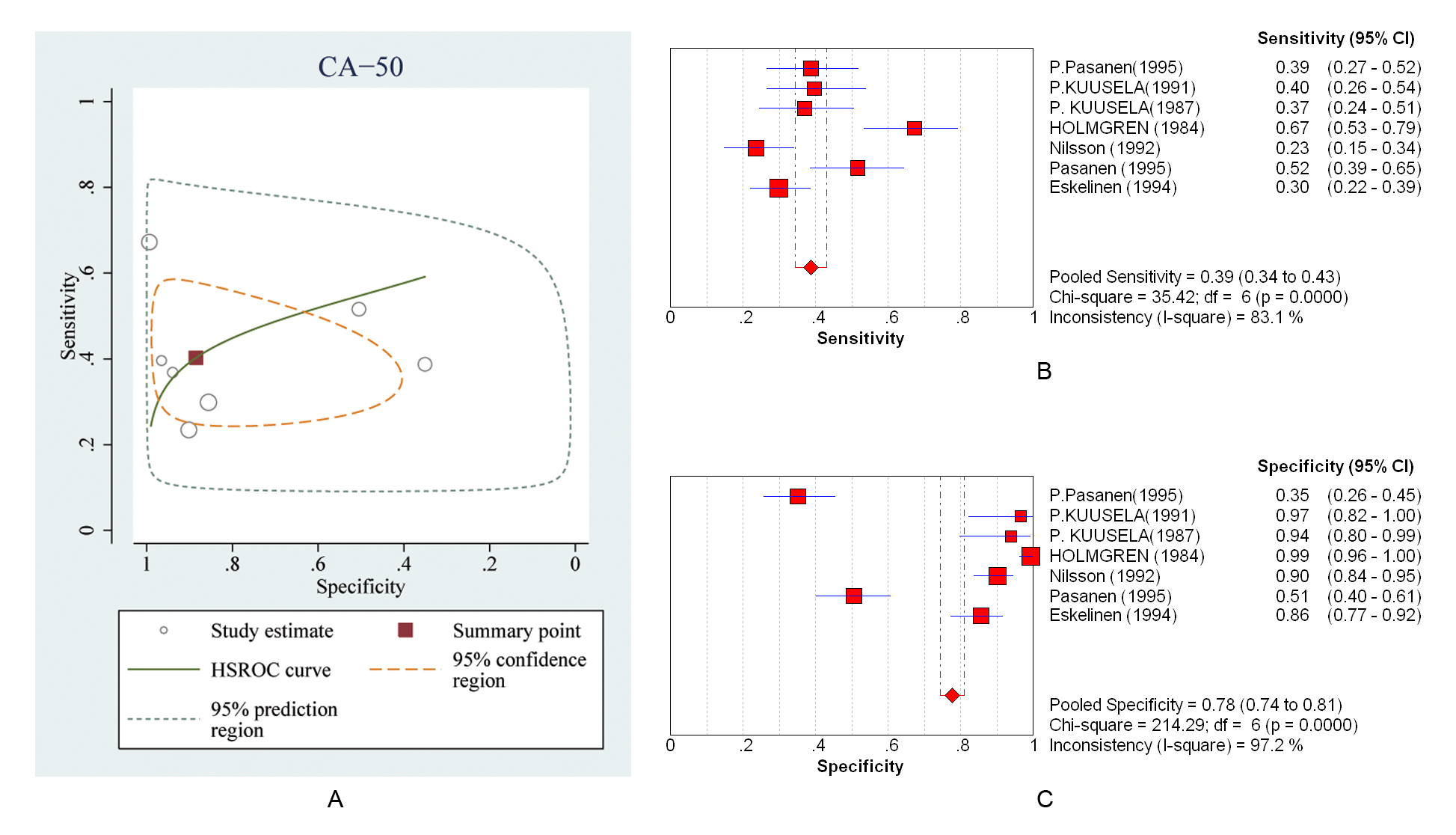


HSROC plot (A), forest plots of specificity (B) and sensitivity (C) of diagnostic marker CA-50 for colorectal cancer

Diagnostic Figure 3S:


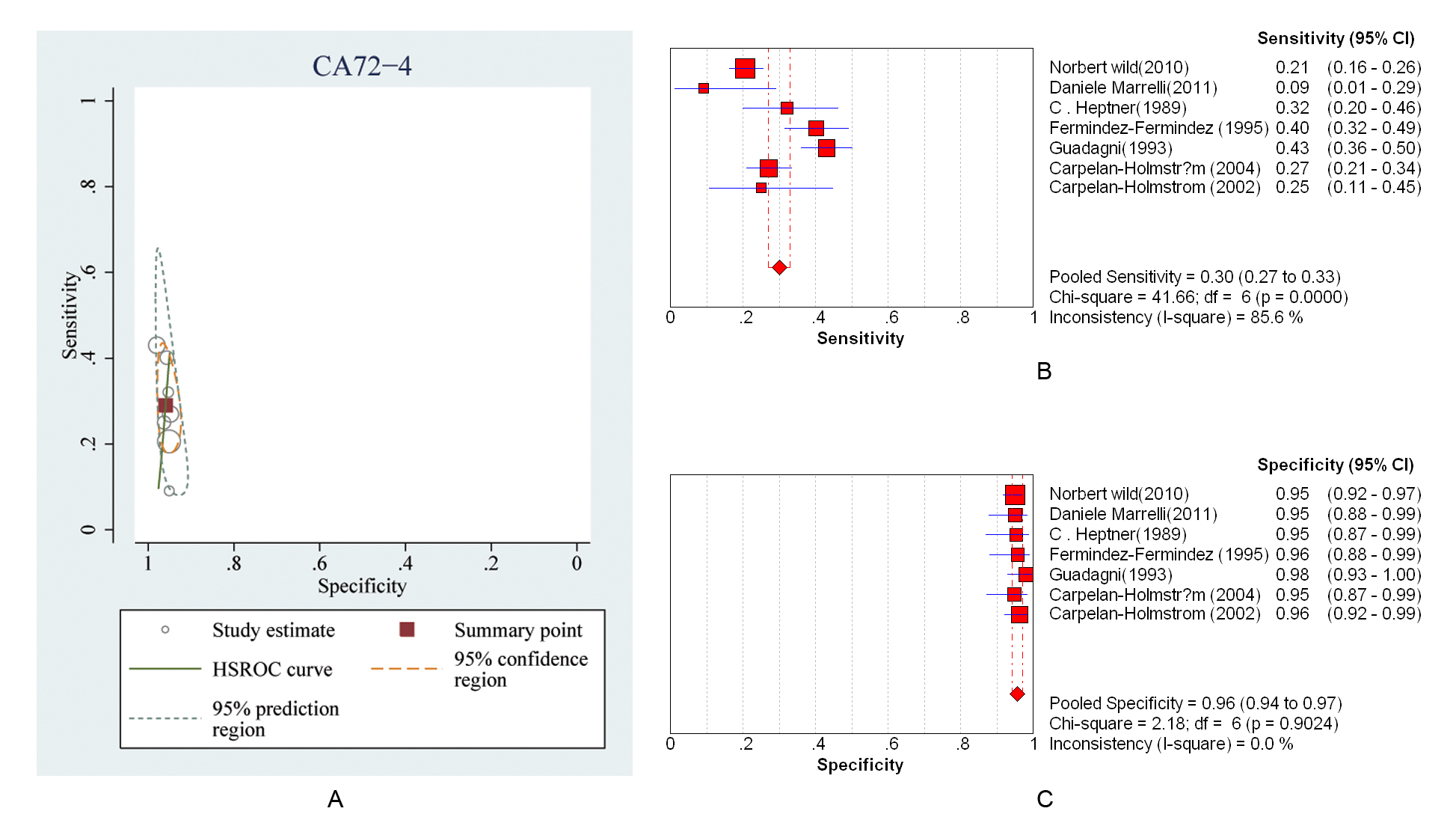


HSROC plot (A), forest plots of specificity (B) and sensitivity (C) of diagnostic marker CA72-4 for colorectal cancer

Diagnostic Figure 4S:


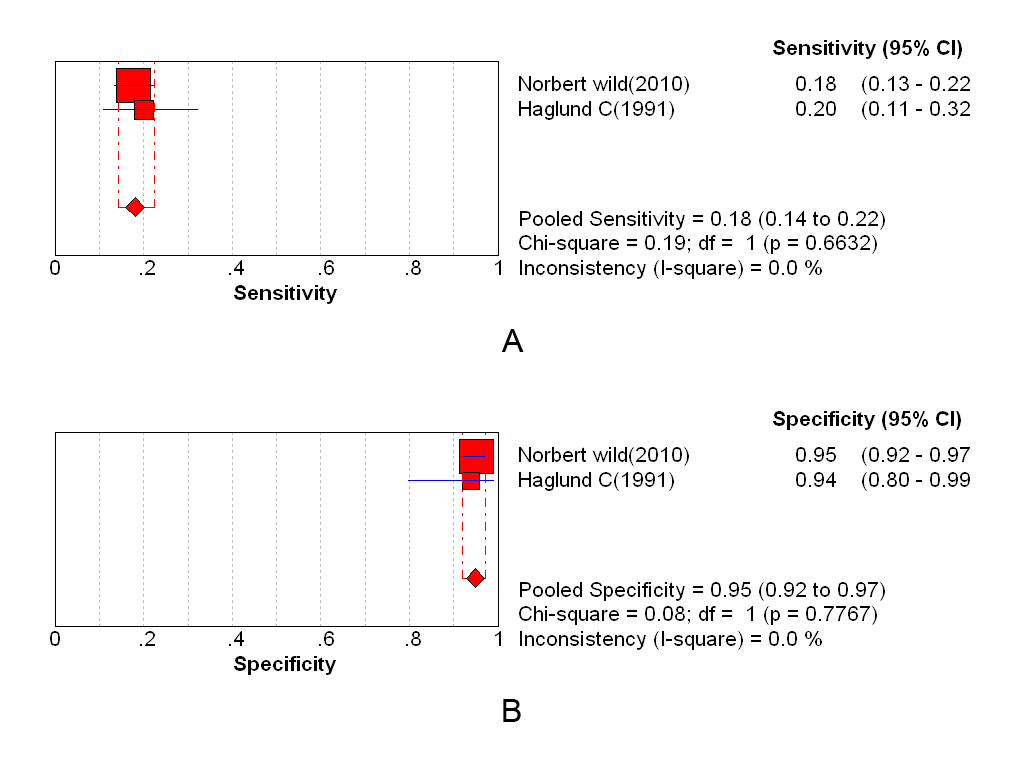


Forest plots of specificity (A) and sensitivity (B) of diagnostic marker CA125 for colorectal cancer

Diagnostic Figure 5S:


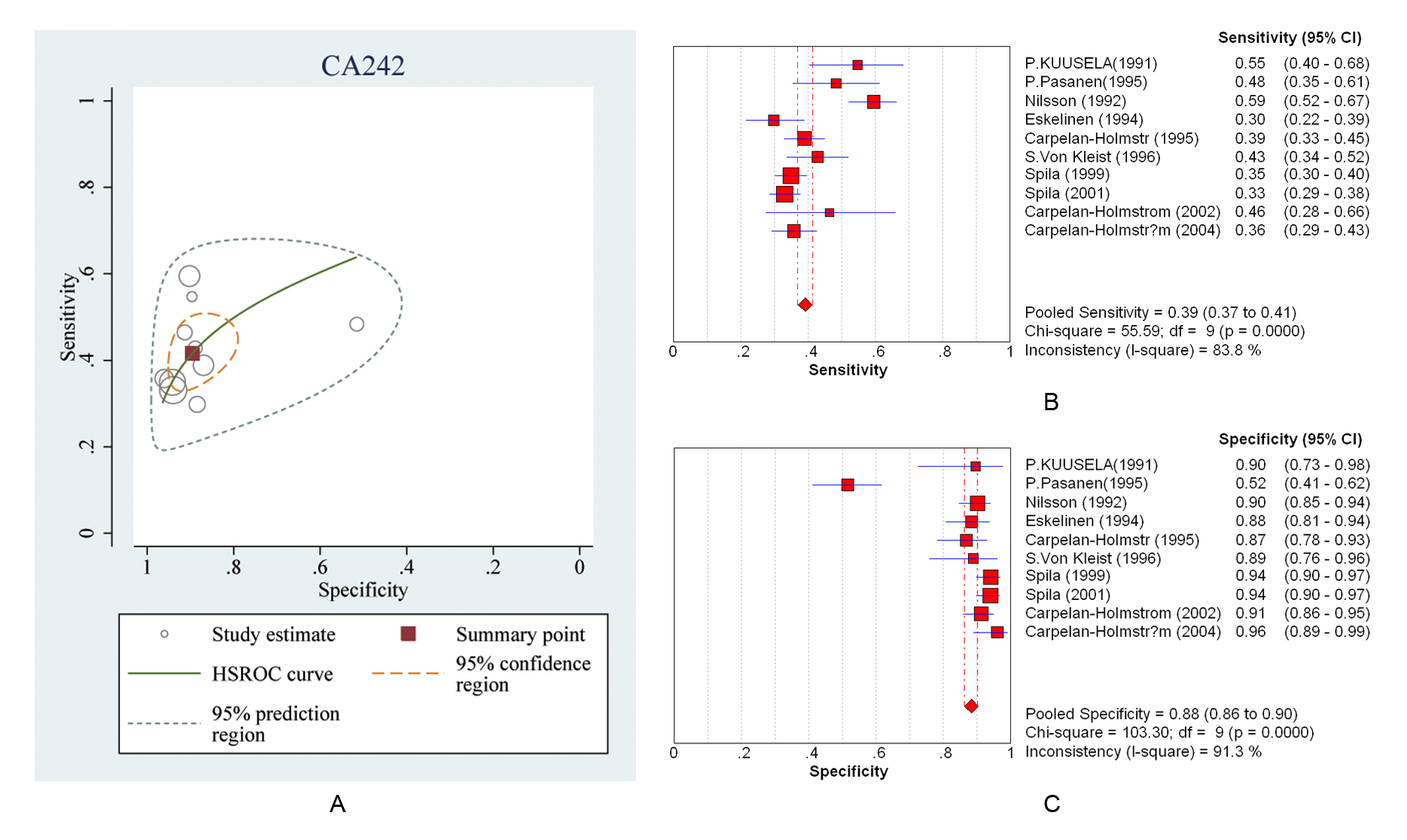


HSROC plot (A), forest plots of specificity (B) and sensitivity (C) of diagnostic marker CA242 for colorectal cancer

Diagnostic Figure 6S:


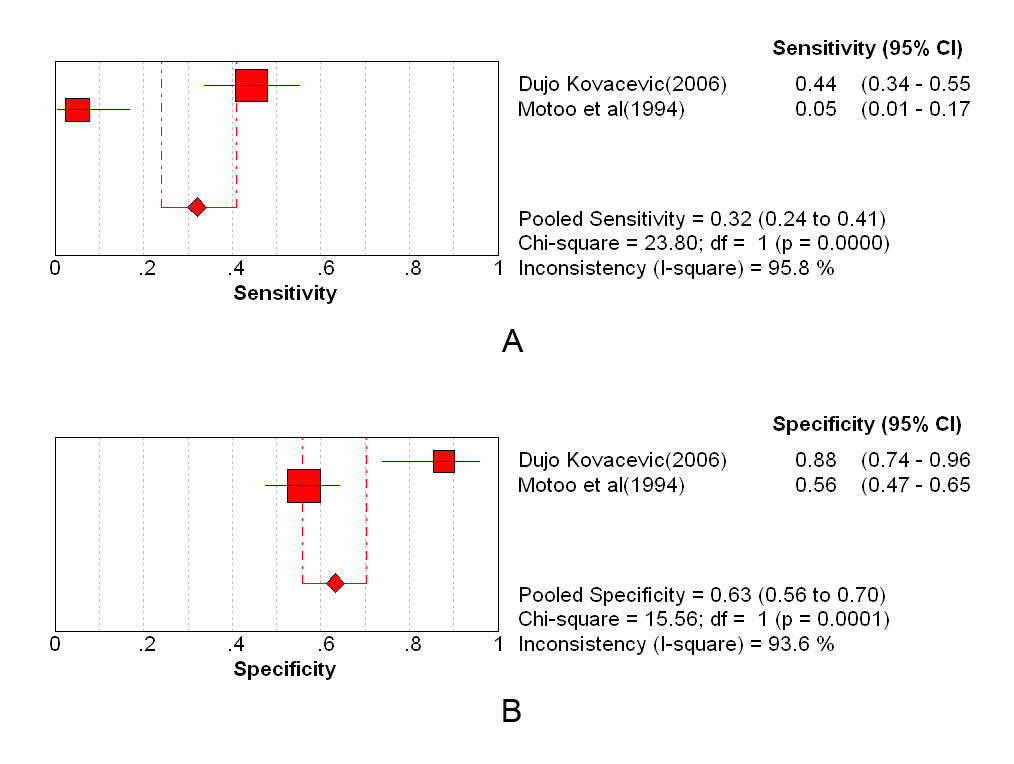


Forest plots of specificity (A) and sensitivity (B) of diagnostic marker c-erbB-2 for colorectal cancer

Diagnostic Figure 7S:


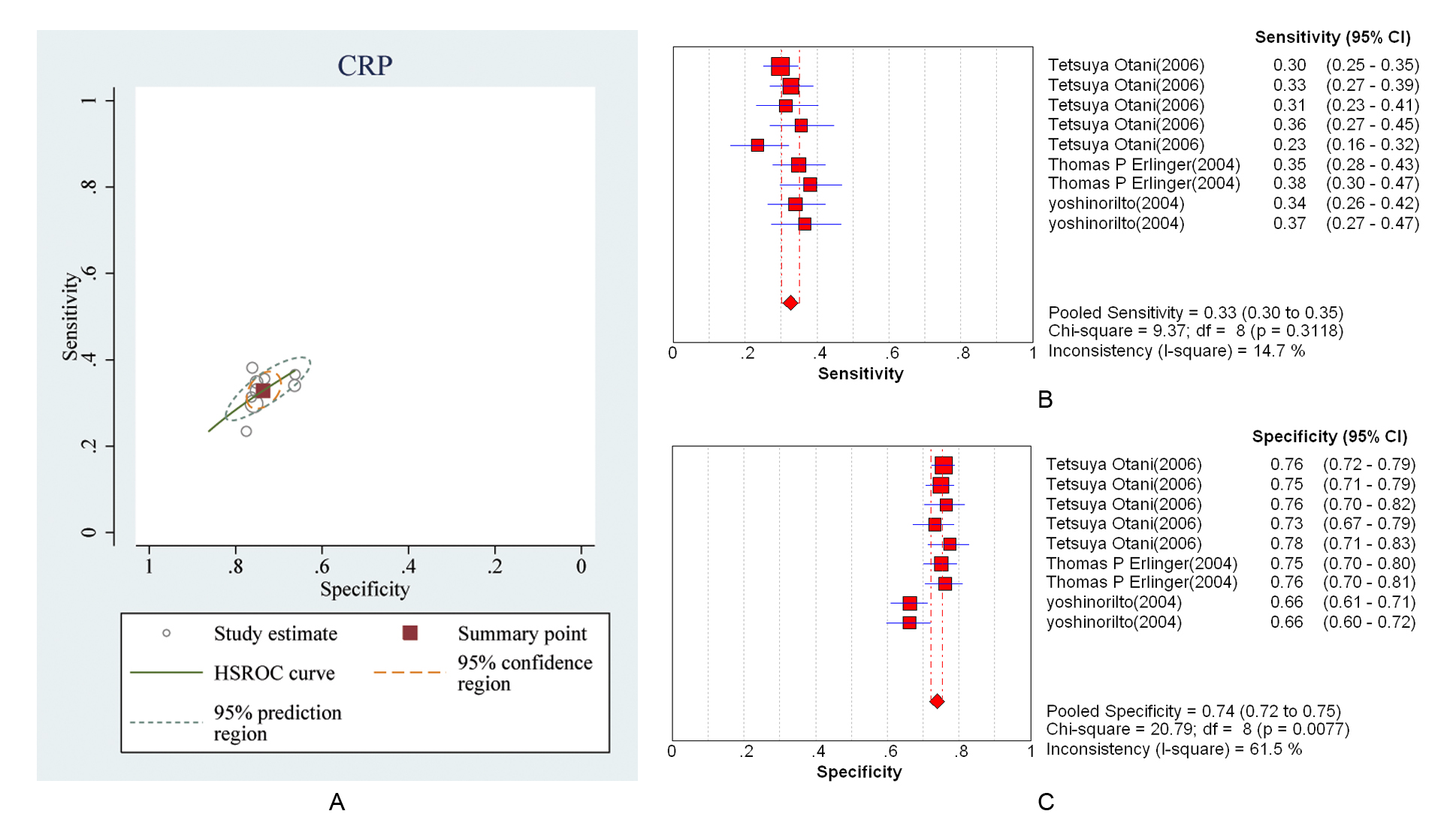


HSROC plot (A), forest plots of specificity (B) and sensitivity (C) of diagnostic marker CRP for colorectal cancer

Diagnostic Figure 8S:


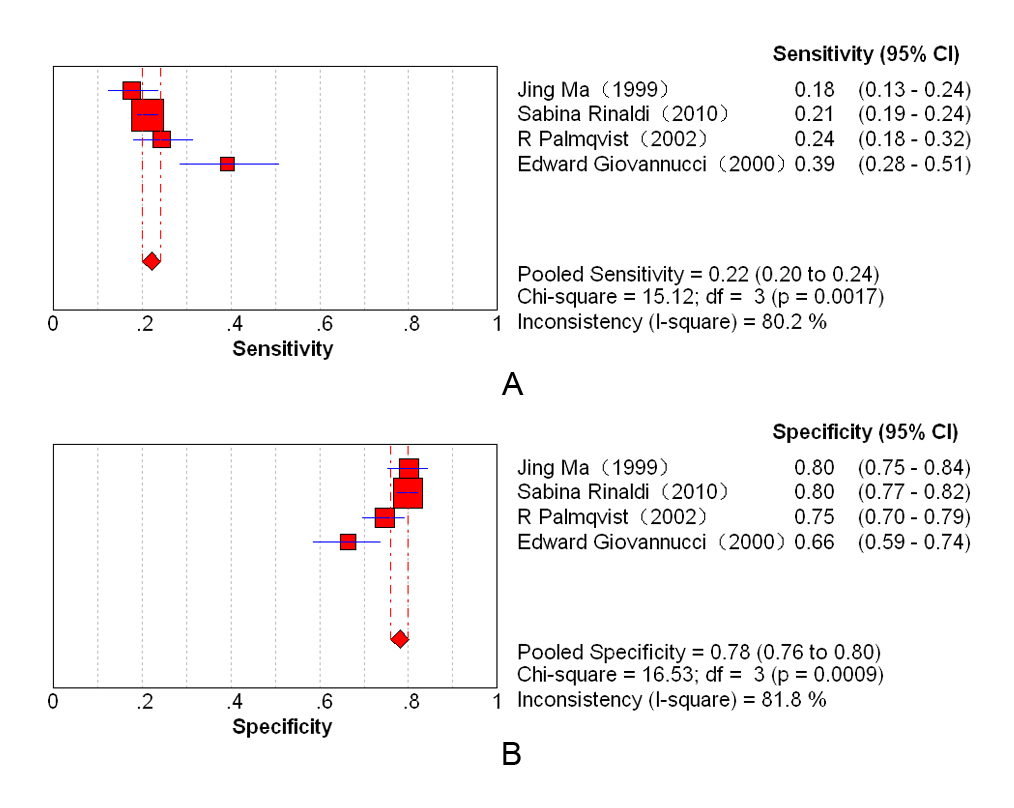


Forest plots of specificity (A) and sensitivity (B) of diagnostic marker IGF-1 for colorectal cancer

Diagnostic Figure 9S:


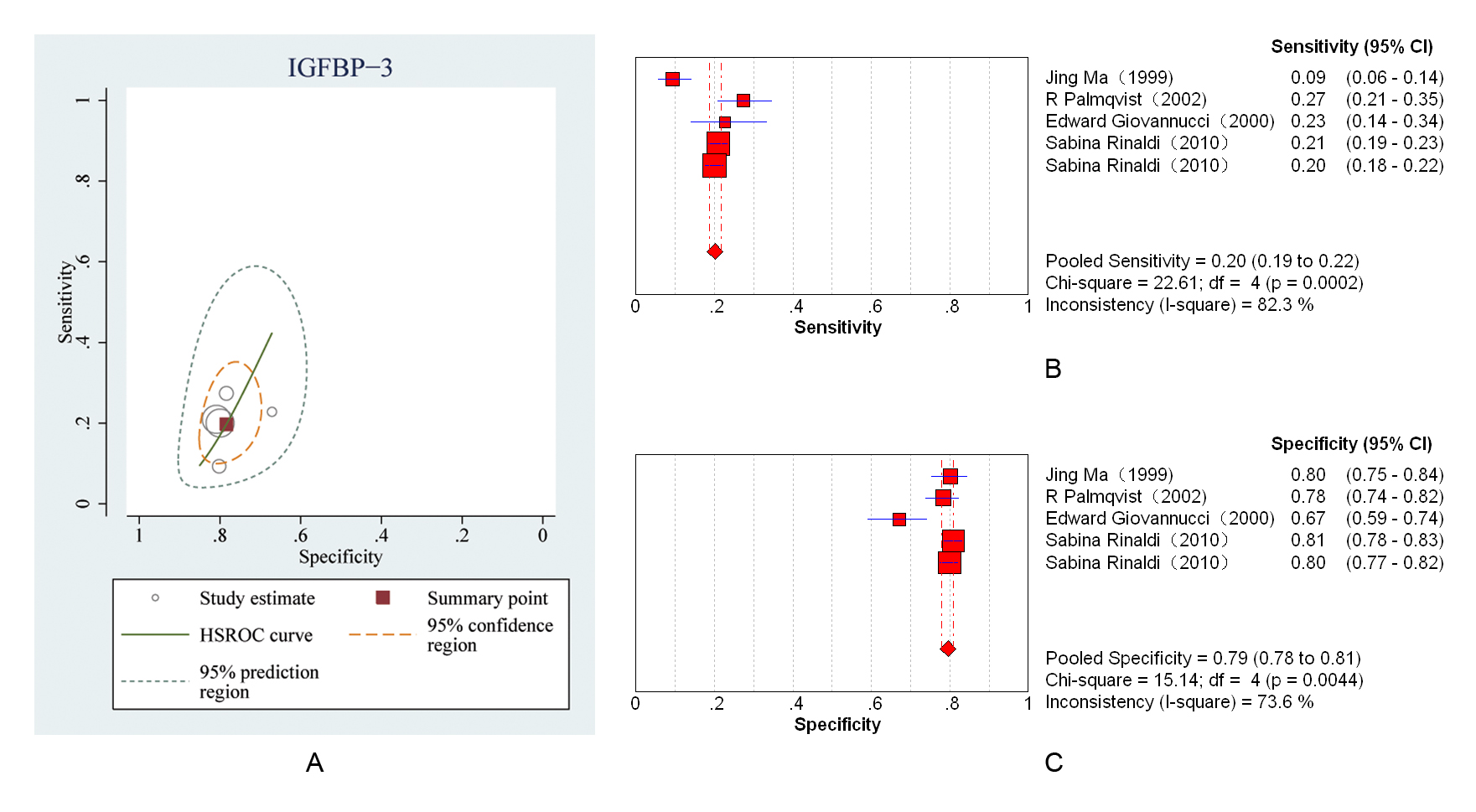


HSROC plot (A), forest plots of specificity (B) and sensitivity (C) of diagnostic marker IGFBP-3 for colorectal cancer

Diagnostic Figure 10S:


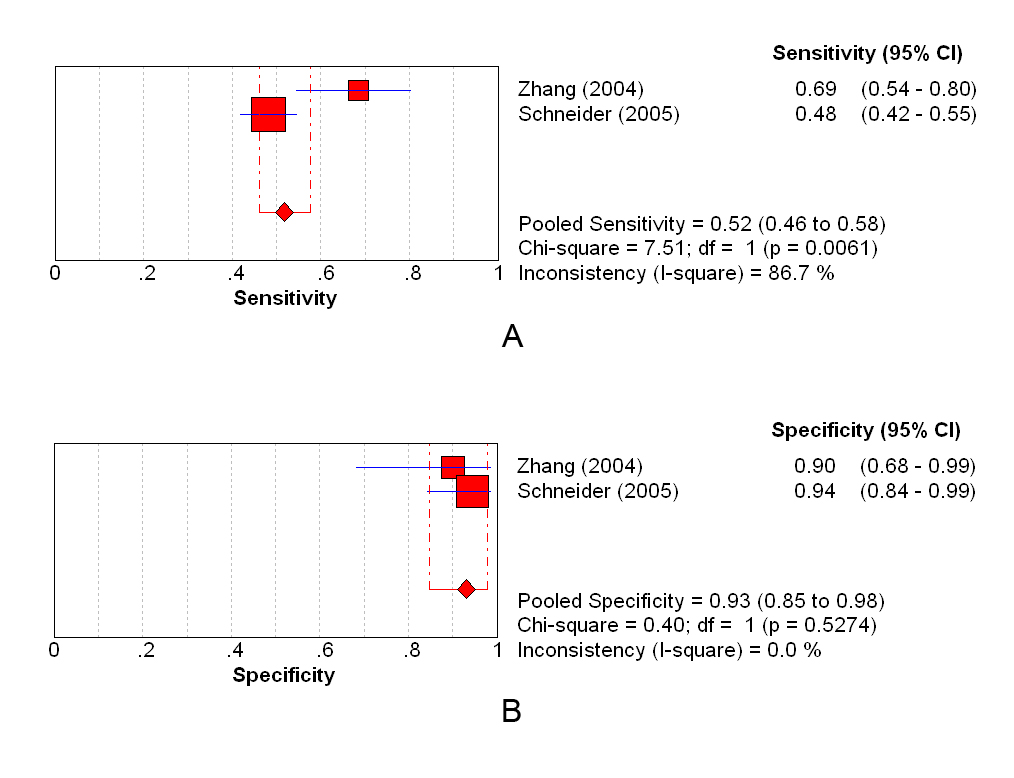


Forest plots of specificity (A) and sensitivity (B) of diagnostic marker M2-PK for colorectal cancer

Diagnostic Figure 11S:


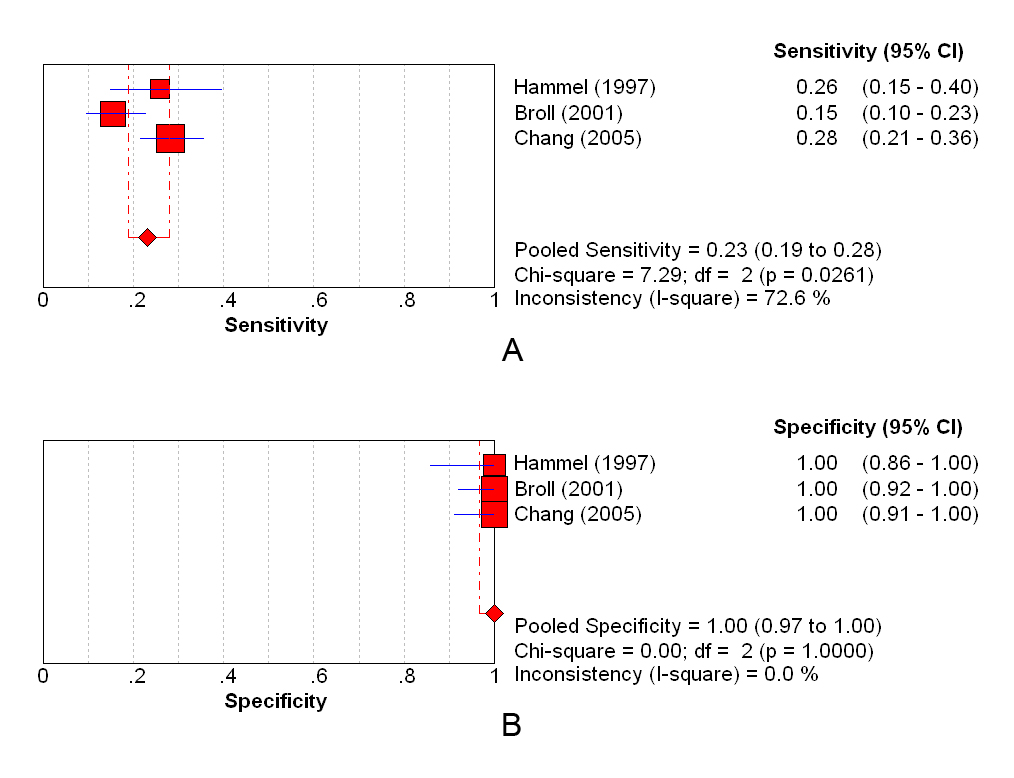


Forest plots of specificity (A) and sensitivity (B) of diagnostic marker P53 for colorectal cancer

Diagnostic Figure 12S:


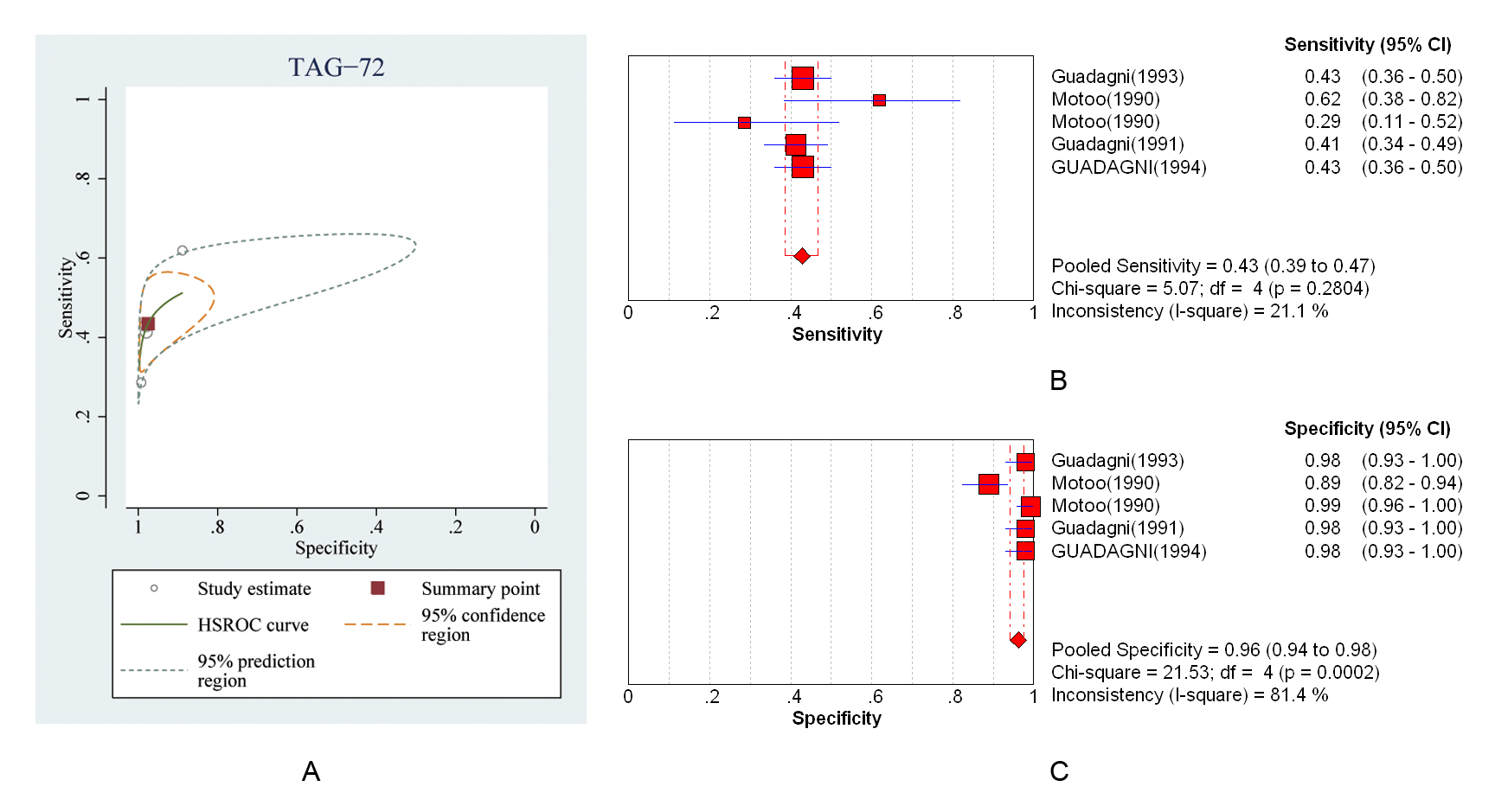


HSROC plot (A), forest plots of specificity (B) and sensitivity (C) of diagnostic marker TAG-72 for colorectal cancer

Diagnostic Figure 13S:


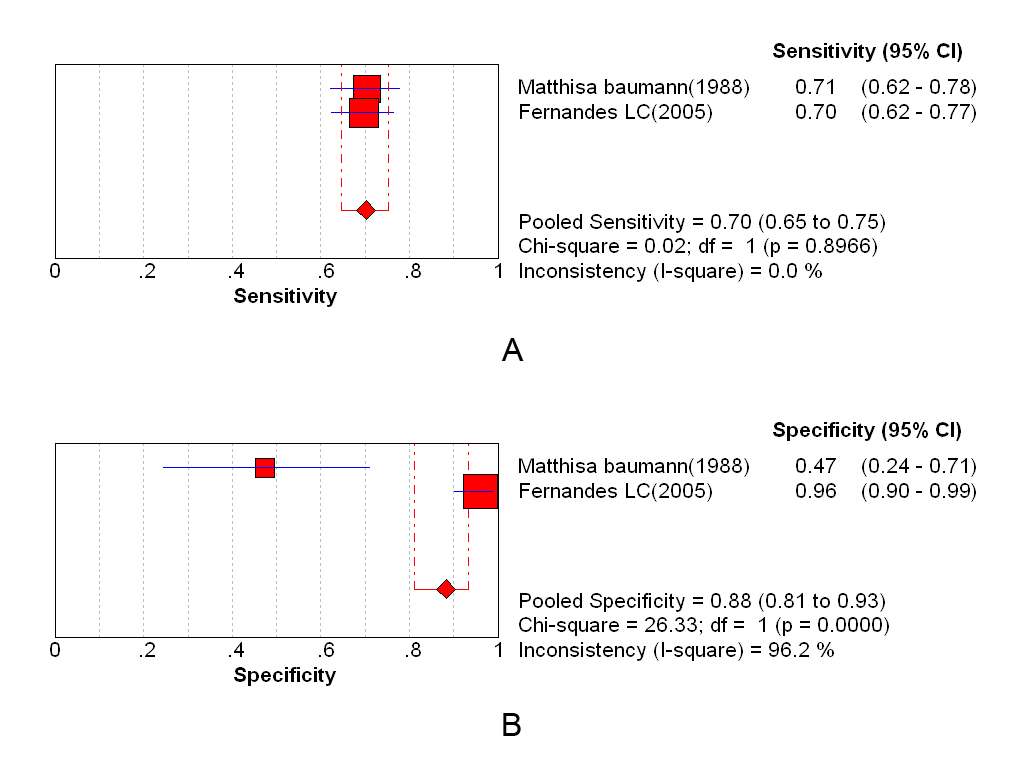


Forest plots of specificity (A) and sensitivity (B) of diagnostic marker TPA-M for colorectal cancer

Diagnostic Figure 14S:


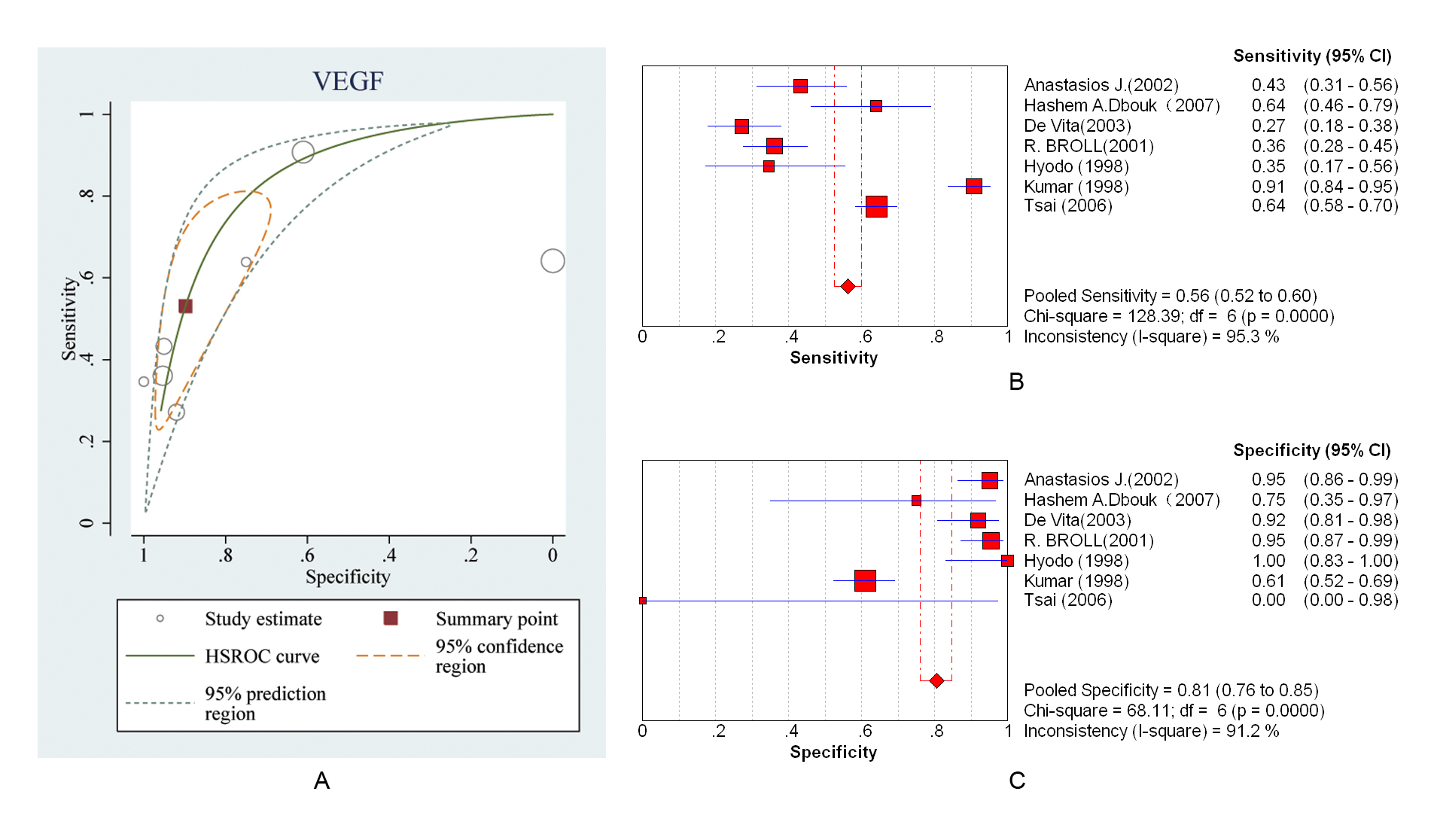


HSROC plot (A), forest plots of specificity (B) and sensitivity (C) of diagnostic marker VEGF for colorectal cancer

# Appendix 7

The funnel plots with “missing ” studies of Prognostic markers having enough evidences of publication bias . A circle denotes a study and a square inside a circle denotes a “missing” study.

**CEA**

**CA19-9**

**CA242**

**CRP**

**VEGF**

**CA50**

**CA72-4**

**IGFBP-3**

**TAG-72**

**IGF-1**

**CA125**

**c-erbB-2**

**TPA-M**

**TIMP-1**

**M2-PK**

# Appendix 8

Prognostic markers supplementary figures

Prognostic Figure 1S:


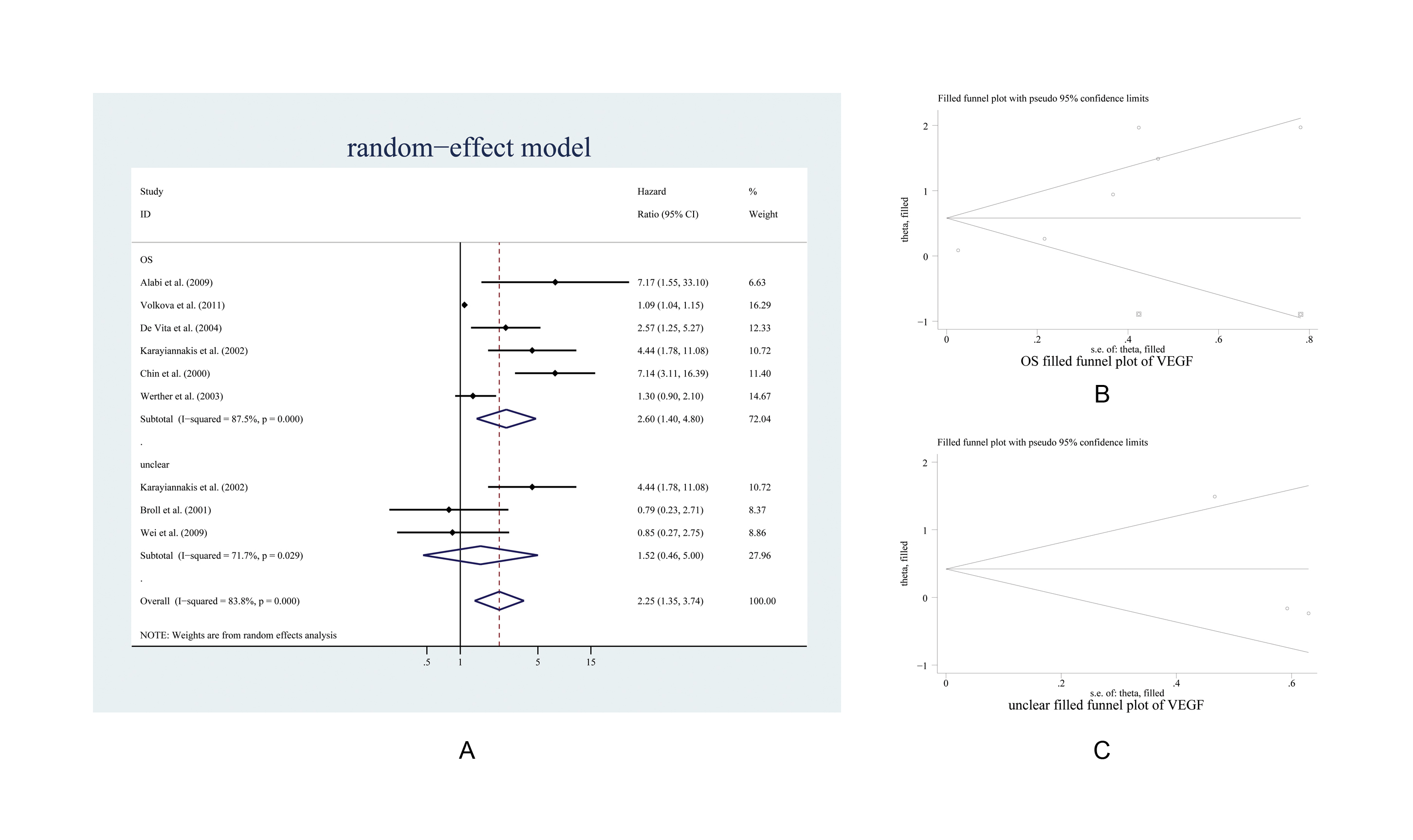


Forest plot of (A), filled funnel plots of OS (B) and unclear (C) subgroups of prognostic marker VEGF of colorectal cancer

Prognostic Figure 2S:


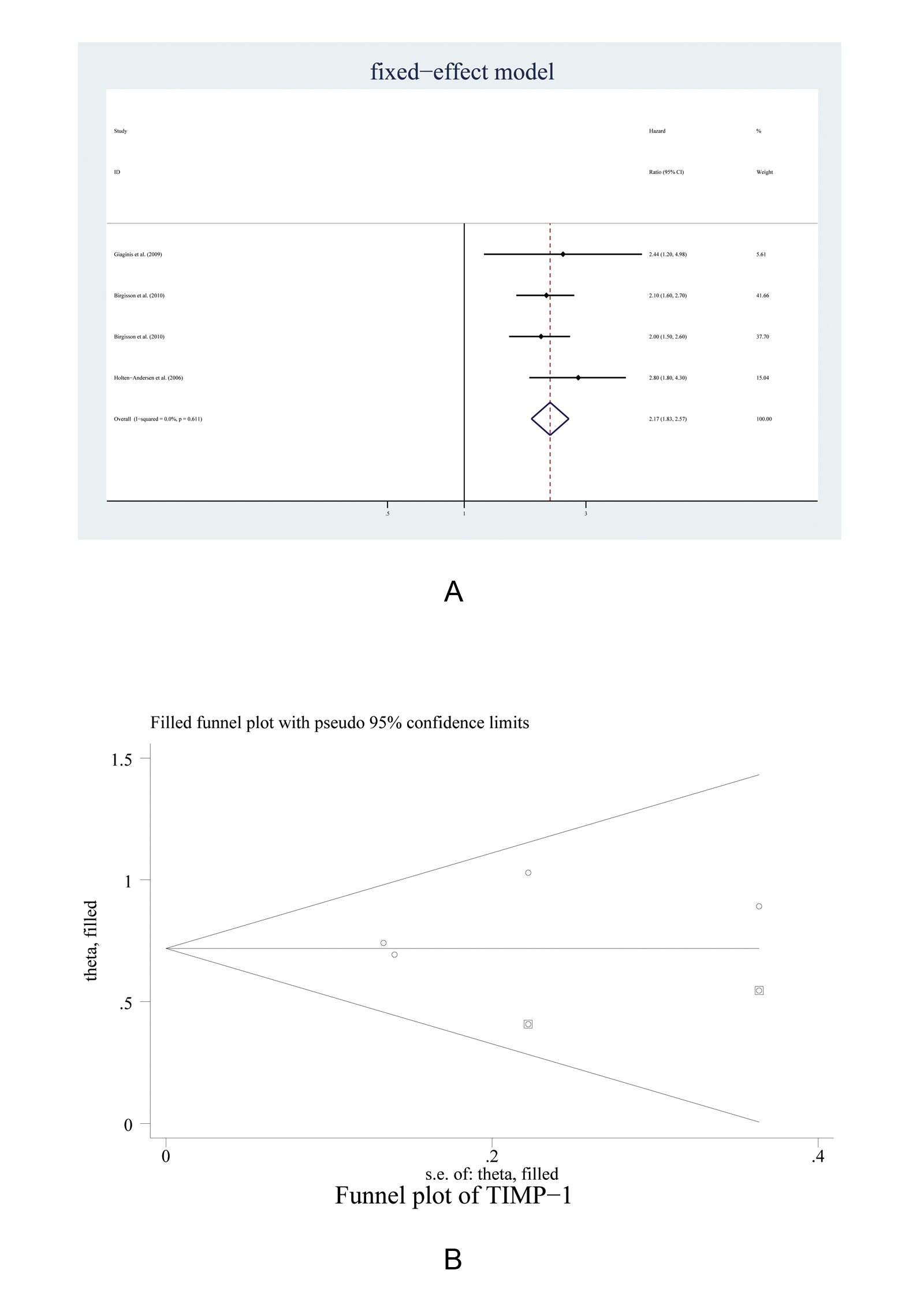


Forest plot of (A), filled funnel plots (B) of prognostic marker TIMP-1 of colorectal cancer

Prognostic Figure 3S:


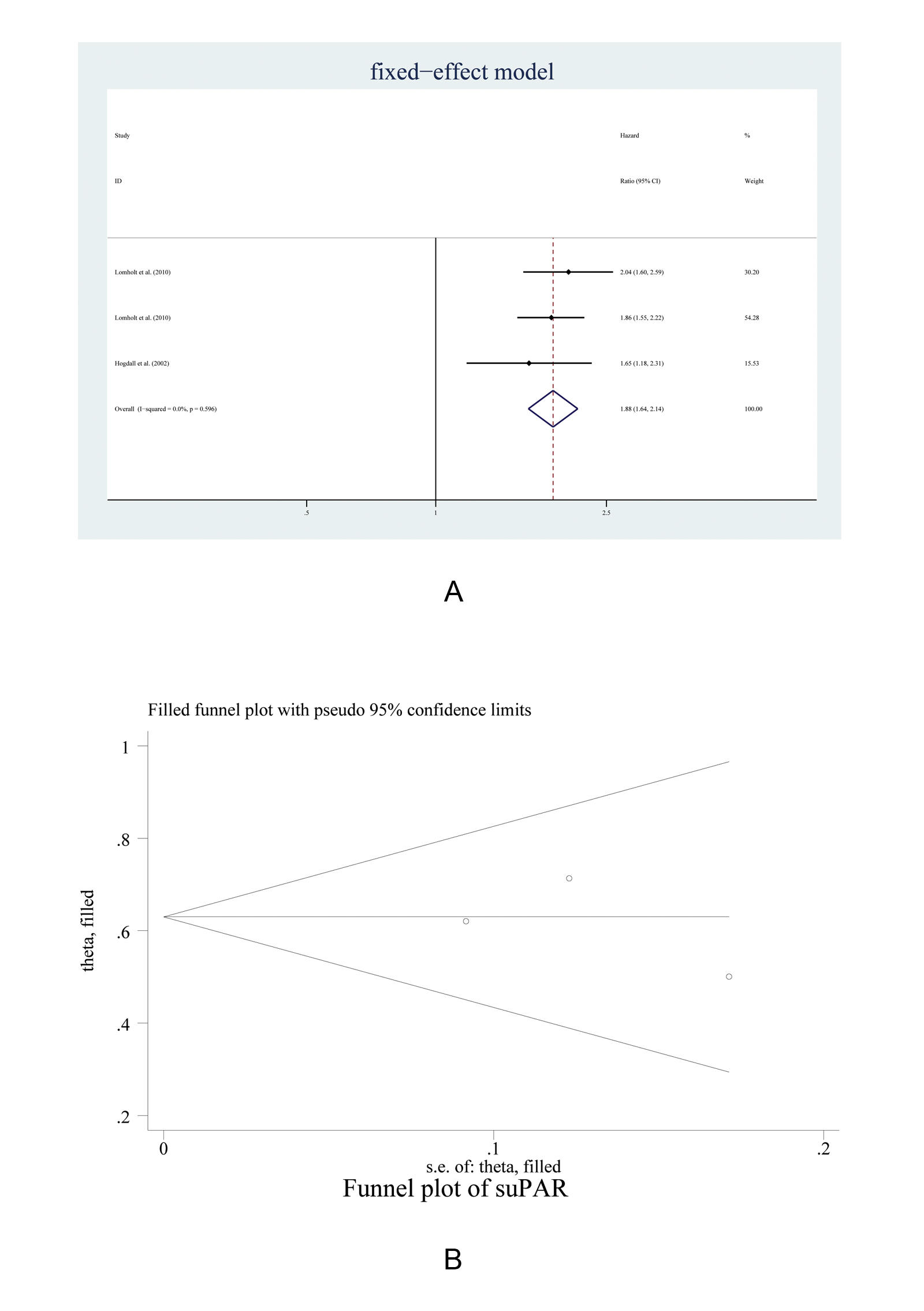


Forest plot of (A), filled funnel plots (B) of prognostic marker suPAR of colorectal cancer

Prognostic Figure 4S:


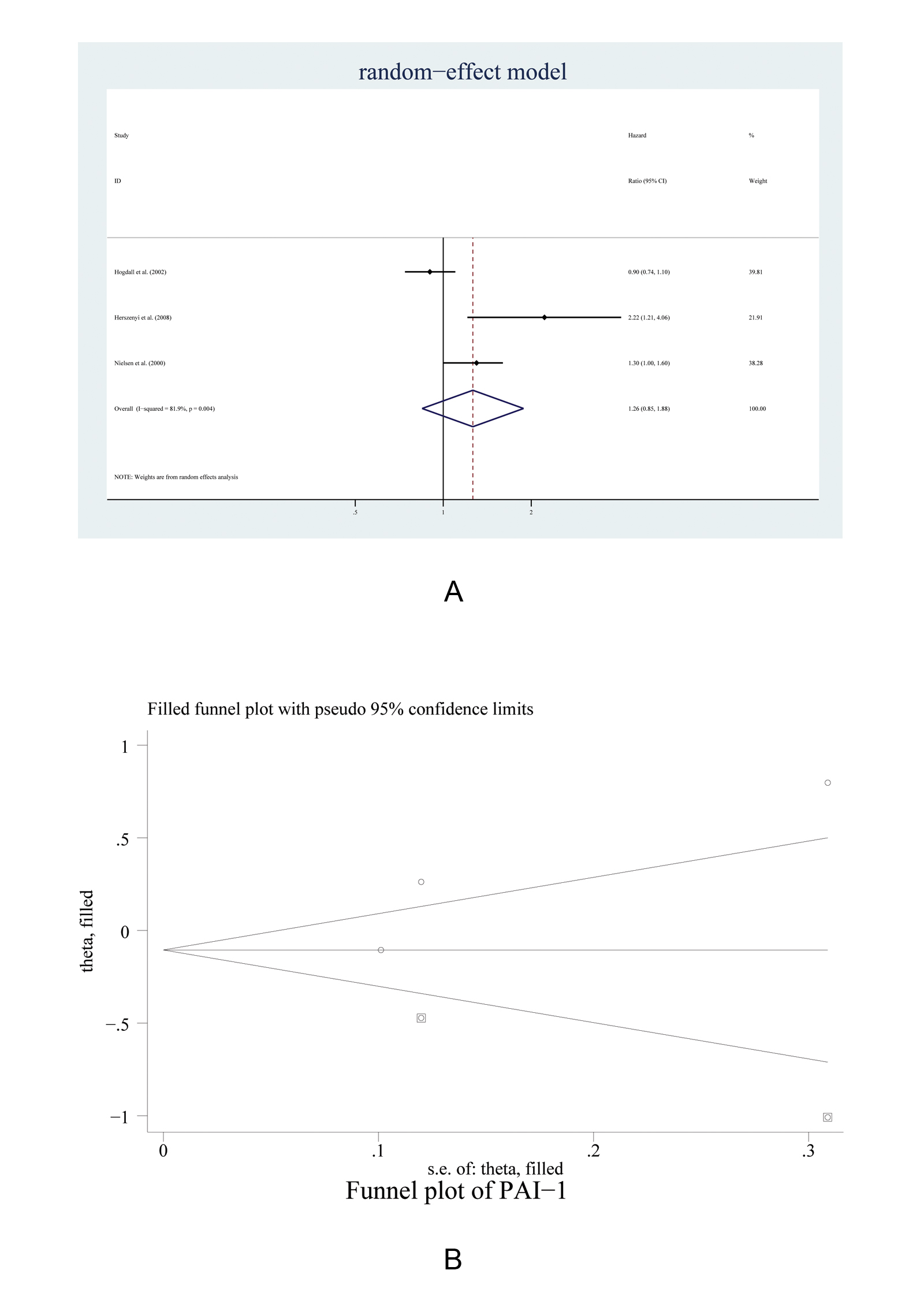


Forest plot of (A), filled funnel plots (B) of prognostic marker PAI-1 of colorectal cancer

Prognostic Figure 5S:


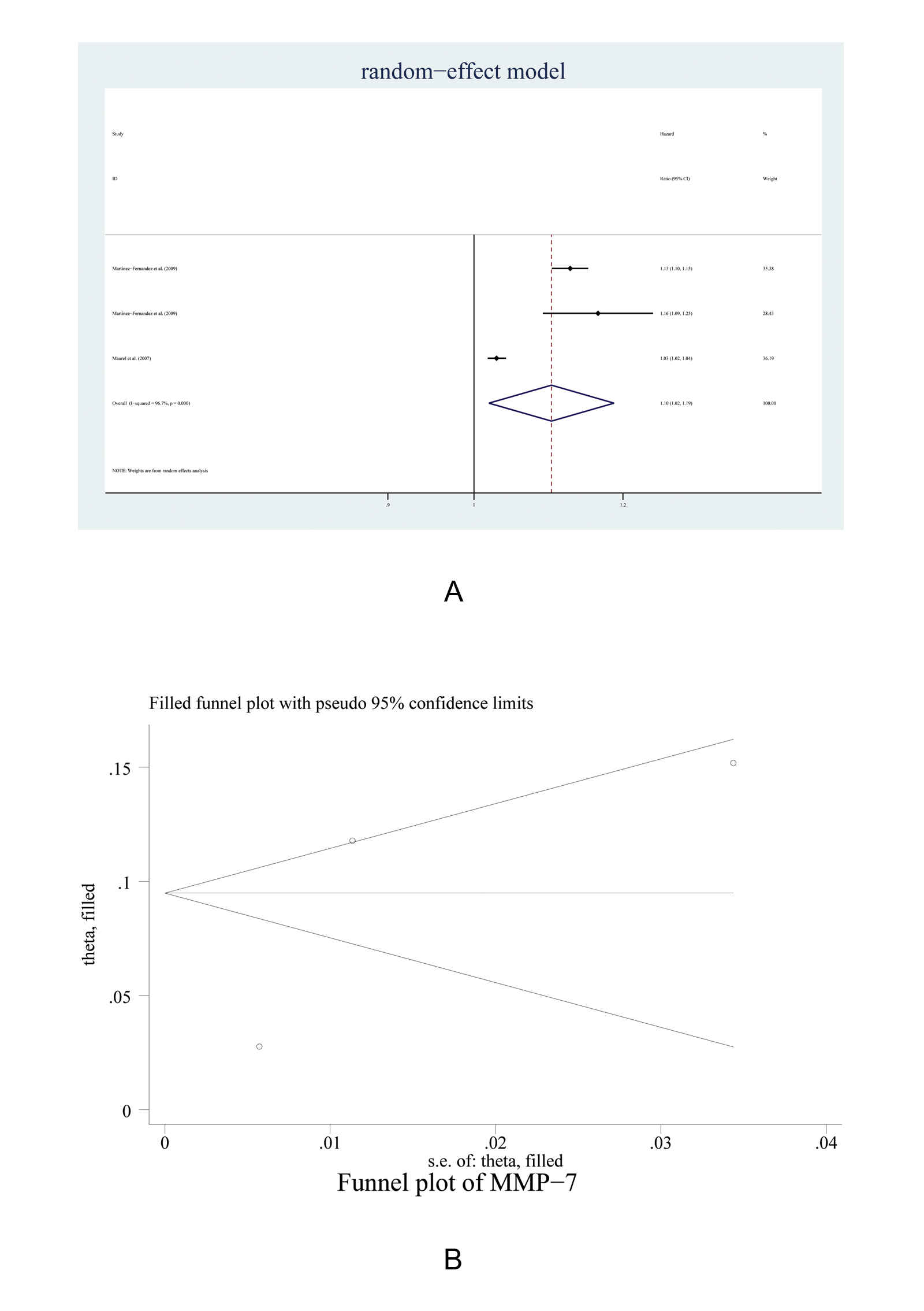


Forest plot of (A), filled funnel plots (B) of prognostic marker MMP-7 of colorectal cancer

Prognostic Figure 6S:


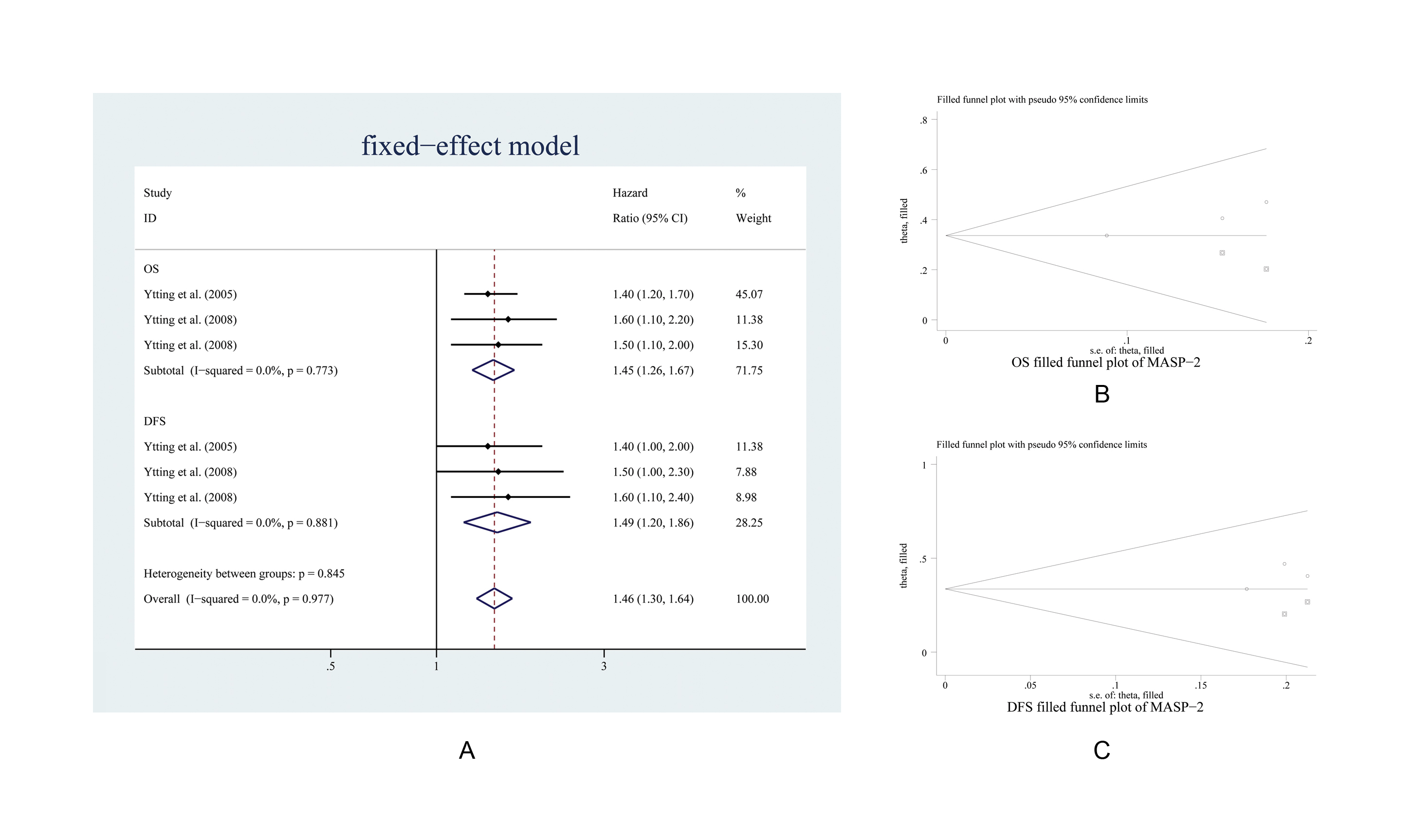


Forest plot of (A), filled funnel plots of OS (B) and DFS (C) subgroups of prognostic marker MASP-2 of colorectal cancer

Prognostic Figure 7S:


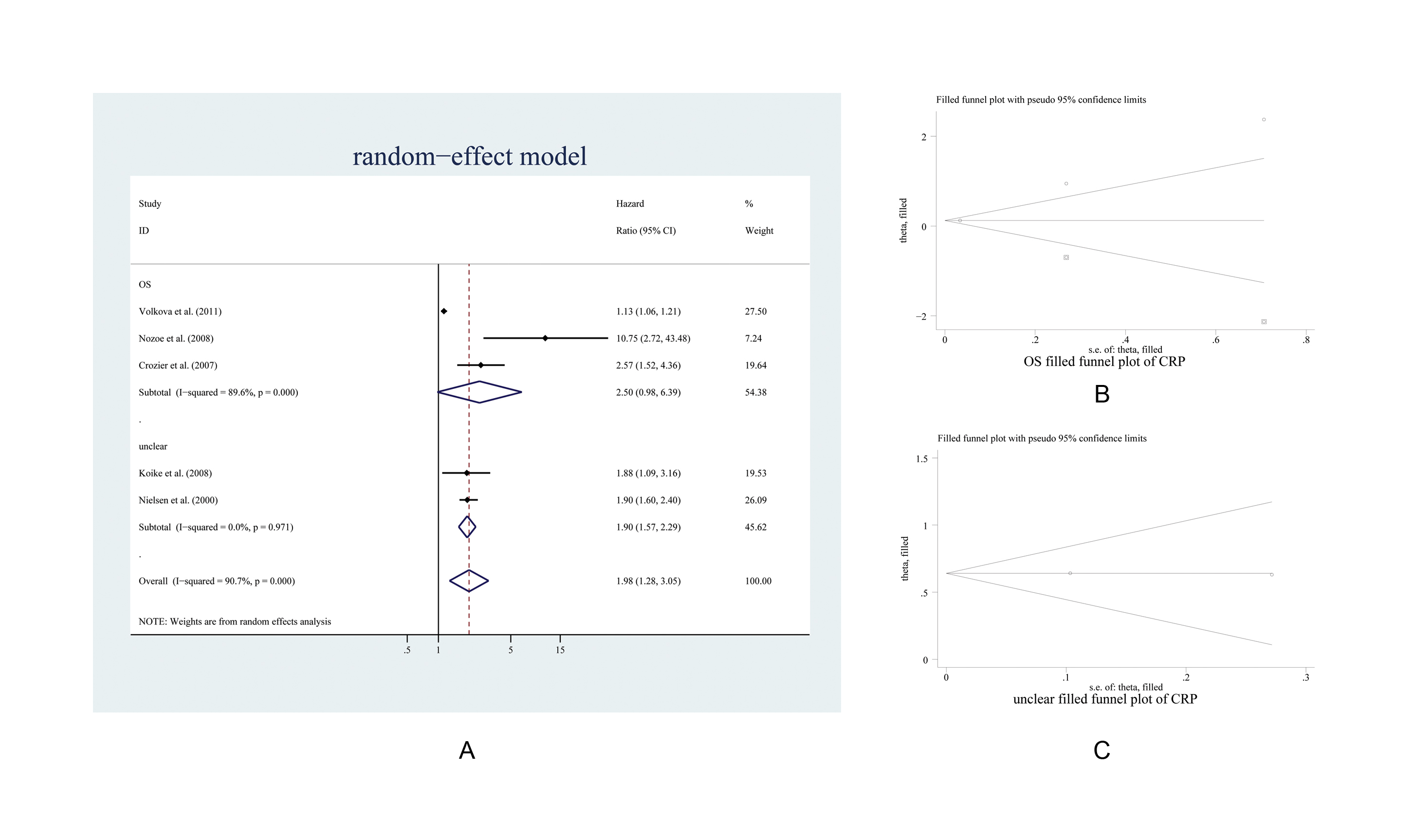


Forest plot of (A), filled funnel plots of OS (B) and unclear(C) subgroups of prognostic marker CRP of colorectal cancer

Prognostic Figure 8S:


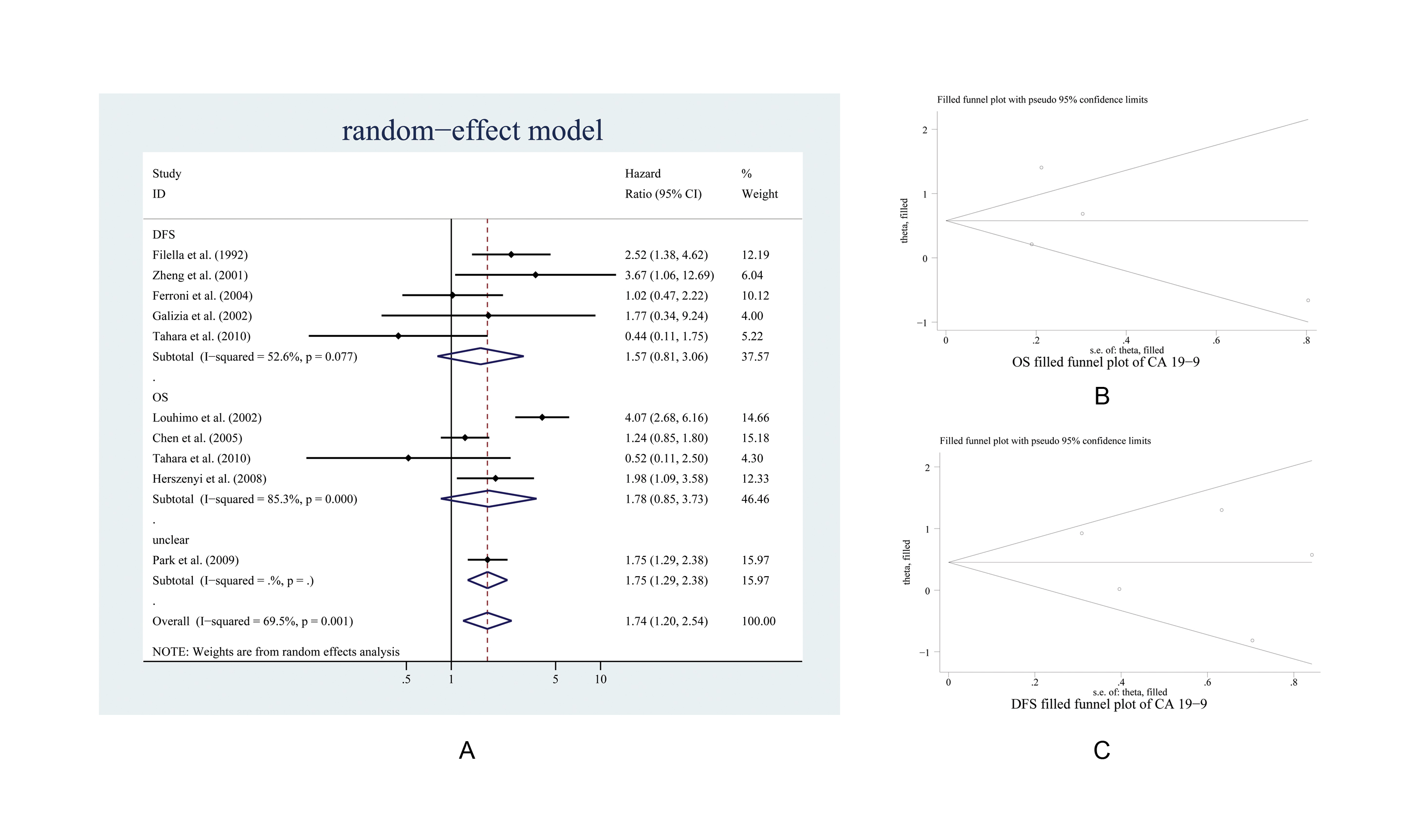


Forest plot of (A), filled funnel plots of OS (B) and DFS(C) subgroups of prognostic marker CA 19-9 of colorectal cancer

Prognostic Figure 9S:


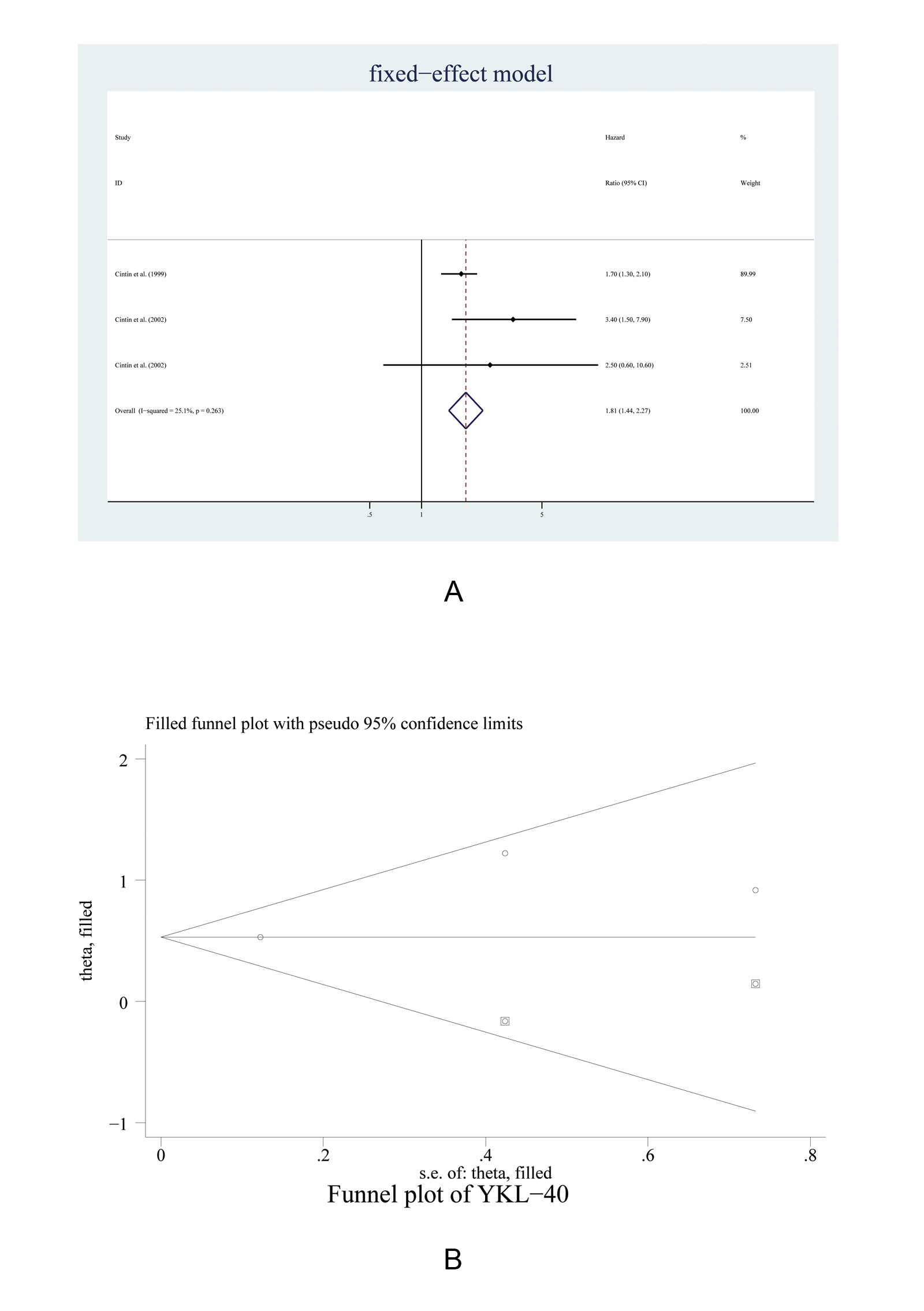


Forest plot of (A), filled funnel plots of OS (B) prognostic marker YKL-40 of colorectal cancer
